# Recruitment of beneficial cucumber rhizosphere microbes mediated by amino acid secretion induced by biocontrol *Bacillus subtilis* isolate 1JN2

**DOI:** 10.3389/fmicb.2024.1379566

**Published:** 2024-04-04

**Authors:** Wei Yang, Xiao Li, Haixia Yan, Yiwen Sun, Diwen Wu, Ying Du, Yuming Luo

**Affiliations:** ^1^Jiangsu Key Laboratory for Eco-Agricultural Biotechnology Around Hongze Lake, Jiangsu Collaborative Innovation Center of Regional Modern Agriculture and Environmental Protection, Huaiyin Normal University, Huai’an, China; ^2^Agro-Tech Extension and Service Center, Huai’an, China

**Keywords:** *Fusarium* wilt, biocontrol, recruitment, amino acids, rhizosphere microbiome

## Abstract

**Introduction:**

At present, the use of beneficial microorganisms to control cucumber *Fusarium* wilt is a widely used method, and the rhizosphere microecological reset is one of the mechanisms involved. However, how biocontrol strains reshape cucumber rhizosphere microecology remains to be further studied.

**Methods:**

The composition changes of cucumber root exudates induced by biocontrol strain 1JN2, the microbial ecology of cucumber rhizosphere and the colonization ability of biocontrol strain 1JN2 in cucumber rhizosphere were analyzed through UHPLC-MS/MS analysis, Illumina high-throughput sequencing and SEM, respectively.

**Results:**

First, cucumber plants treated with biocontrol *Bacillus* 1JN2 reduced the disease severity of *Fusarium* wilt by 60%. Significant changes in cucumber root exudates were found after 1JN2 inoculation and the contents of four amino acids including glutamine, tryptophan, glycine and glutamic acid were significantly increased. Second, It was found that the bacterial diversity in the rhizosphere of cucumber was significantly increased in both the strain treatment group and the amino acid mixture treatment group, The number of *Bacillus* was the largest in all dominant populations, exceeded 20% in all treatment groups. The bacteria of *Hydrogenispora* and Vicinamibacteria were significantly increased after treatment.

**Discussion:**

Overall, the results demonstrated that amino acid substances in cucumber root exudates induced by biocontrol strain 1JN2 can shift the cucumber root microenvironment and prevent the occurrence of *Fusarium* wilt disease.

## 1 Introduction

Cucumber is one of the vegetables that widely cultivated worldwide. *Fusarium* wilt of cucumber is one of the most destroyed soil-borne diseases caused by *Fusarium oxysporum* (Qiu et al., 2012). At present, the control methods of cucumber withering include agricultural control, chemical control, disease-resistant breeding, and biological control. Biological control uses antagonistic organism, and its metabolites are well accepted due to environmental friendliness (Raza et al., 2017). Among the fungi, *Trichoderma harzianum* and *Trichoderma viride* have reported to be effective biocontrol agents. Liu et al. (2012) reported that *T. harzianum* TN inoculation significantly increased the expression of defense-related genes, such as *G6PDH*, *PAL*, *C4H*, *CCOMT*, *CAD,* and *PR-1* in cucumber, and the activities of dehydrogenase and polyphenol oxidase to inhibit *Fusarium* infection. The chitinase and cellulase produced by *T. viride* can effectively degrade the cell wall of *F. oxysporum*. Among the bacteria, the biocontrol bacteria with good effect of inhibiting *Fusarium* wilt of cucumber include *Bacillus pumilus* and *Bacillus cereus*, which can improve the stress resistance of plants by seizing nutrients from pathogens (Raza et al., 2017). Some actinomycetes can inhibit the mycelial growth and spore development of the pathogen, promote the germination of cucumber seeds, and induce system resistance (Lu et al., 2016).

For soil borne diseases, the use of biocontrol strains is generally limited to plant roots, and the first step for it to show its biocontrol potential is its own colonization, which is also one of the problems that have been puzzling the research of biological control (Berendsen et al., 2012). The factors affecting the colonization of biocontrol strains include not only the interaction between biocontrol strains and host plants but also the interaction between biocontrol strains and pathogen and other microorganisms in plant rhizosphere. First, the habitat from where the biocontrol bacteria was isolated will affect its colonization ability in specific plant rhizosphere. Different plant species and soil types are the main factors that determine the microbial species in plant rhizosphere (Berg and Smalla, 2009). Second, in the plant rhizosphere, another factor affecting the colonization of biocontrol strains is the influence of indigenous populations. Due to the existence of root exudates, in the niche of plant rhizosphere, the competition between various microorganisms is very fierce, and there is a relationship between biocontrol strains, pathogens, and other strains, which has no direct effect on plants (Wen et al., 2020). Therefore, by adjusting the population structure of plant rhizosphere microorganisms, the pathogen can be inhibited, which is also the key to realize the sustainable prevention and control of diseases.

During the interactions between biocontrol agents, pathogens, and indigenous organisms, root exudates from host plant play a key role. Through rhizodeposition and enrichment with energy and carbon sources, plant roots are primarily responsible for the assembly of the rhizosphere microbial composition (Bais et al., 2006). The difference in the microbial community in rhizosphere soil and bulk soil explains the role of host root exudates in shaping rhizosphere microbiome. The release of readily available energy and carbon in different forms, including amino acids, carbohydrates, and sugars, helps the beneficial microbes in the rhizosphere (Berendsen et al., 2012). These forms, in turn, perform crucial tasks, such as nitrogen fixation and nutrient solubilization and disease prevention (Nuccio et al., 2020). Alternatively, the presence of plant root debris and associated lignin, cellulose, and hemicellulose within the rhizosphere represents the substrate and selection factor of microbial species with the enzymatic potential to degrade such compounds, including phytopathogens (Ling et al., 2022). Thus, it is important to consider both aspects of the micro-environmental conditions in the rhizosphere, which primarily not only facilitate beneficial microbes but also influence phytopathogens.

The root exudates can be considered the tools and messengers by which plants regulate and orchestrate their interactions with the surrounding environment, other plants, and soil microbes. The exudates act as repellents or attractants of specific microbes to shape the rhizo-microbiome composition. The composition and extent of root exudates depend on several plant-related factors, including genotype, developmental stage, health status, and mode of photosynthesis (Feng et al., 2021). Moreover, the secretion of root exudates, especially low molecular weight defense compounds, is tightly regulated in plants and involves a complex process of stimuli-based alternations as a strategy for saving energy. An example is the secretion of certain antimicrobials upon pathogen attack (Baetz and Martinoia, 2014). Defense phytochemicals are synthesized in response to a trigger by pathogens; an example is phytoalexins, such as phenylpropanoids. Other defense compounds (i.e., phytoanticipins, such as the diterpene rhizathalene A) are continuously secreted in the root system, even in the absence of the pathogen (Olanrewaju et al., 2019). These compounds represent the constitutive direct defense of the root system. The absence of the constitutive compounds could indicate higher plant susceptibility to certain pathogens (Van Etten et al., 1994; Badri et al., 2009; Vaughan et al., 2013). Pathogen infection can also trigger higher production of certain constitutive phytoanticipins; an example of the trigger is the increased production of momilactone A by rice plants. These constitutive compounds play a role in the suppression of pathogens and provide a competitive advantage for root establishment (Kato-Noguchi et al., 2008; Hasegawa et al., 2010).

On the other hand, the application of biocontrol strains can also change the composition of root exudates of host plants. In our previous study, we found that the biocontrol strain *B. cereus* AR156 can prevent pathogen infection through SA and JA/ET signaling pathways not only by regulating plant resistance but also by changing the amount of plant root exudates and the composition of rhizosphere microorganisms (Wang et al., 2019). In this study, another biocontrol agent, *Bacillus subtilis* 1JN2, which is isolated from the stem of ginger and has been proven an effective biocontrol agent against *Ralstonia* wilt on ginger and tomato (Yang et al., 2012), was used to control *Fusarium* wilt on cucumber. The composition of the root exudates and microorganisms of cucumber was analyzed, and the amino acids played a key role in the regulation. This study reveals a new interaction between biocontrol agent, cucumber plant, and indigenous microorganisms and provides a new idea for biocontrol agent screening.

## 2 Materials and methods

### 2.1 Bacterial strains, pathogens, plants, and growth conditions

*Bacillus subtilis* 1JN2 was isolated from ginger stem in our previous study and identified as an effective biocontrol agent (GeneBank accession number GU549436，China General Microbiology Culture Collection Center, CGMCC accession number 9759). The strain was grown on LB medium (10 g of tryptone, 5 g of yeast extract, and 5 g of NaCl per liter).

*Fusarium oxysporum* (Schl.) F.sp. *cucumerinum* Owen strain was provided by Kai Chen from Ecology Institute of Shandong Academy of Sciences. The strain was grown on PDA medium (filtrate of 200 g boiled potatoes and 20 g glucose per liter).

Cucumber seeds (Jindian 303, purchased from Academy of Agricultural Sciences of Huai’an, Jiangsu Province, China) were grown in a nursery site, transplanted to a new pot (10 cm height x 10 cm diameter of bottom), and filled with 0.5 kg of nutrient soil composed of vermiculite, peat, and perlite (purchased from Huai’an Chaimihe Agricultural Technology Co., Ltd) when it had 3–4 true leaves.

### 2.2 Greenhouse experiment

Two treatment conditions were tested in the first round greenhouse experiment: A: Blank control, B: *B. subtilis* 1JN2. Each group has three repetitions, and each repetition contains 20 cucumber seedlings. After transplantation of the cucumber plants, suspension of *B. subtilis* 1JN2 was adjusted to 10^7^ CFU/mL, and 20 mL of the suspension was irrigated around the base of the stem for each plant in the group B. After 1 week, 20 mL of spore suspension (10^5^ CFU/mL) of *F. oxysporum* was irrigated for each plant in the two groups. The temperature of the greenhouse was maintained at 30°C with 16/8 h of light/dark light cycle. The disease index was recorded 30 days after transplanting.

### 2.3 Analysis of root colonization by *Bacillus subtilis* 1JN2

For colonization analysis, a chloramphenicol-resistant mutant 1JN2-chl was screened using methods from the study by Xue (2008) and prepared in LB medium with 20 μg/mL of chloramphenicol. As described above, a second-round greenhouse experiment was performed with two groups: A: cucumber, B: nutrient soil without plant. Each group has three repetitions, and each repetition contains 10 cucumber seedlings. After transplantation of the cucumber plants, suspension of *B. subtilis* 1JN2-chl was adjusted to 10^8^ CFU/mL, and 20 mL of the suspension was irrigated for each plant. In total, 1 g of rhizosphere soil from each group was collected on 1, 3, 7, 14, and 21 days after treatment. The colonization ability of the strain was measured by dilution spreading on solid medium with 20 μg/mL of chloramphenicol.

For scanning electron microscopy analysis, 20 cucumber seedlings with 4–5 true leaves were selected and divided into two groups. First, the roots of the plants were washed with sterile distilled water thrice and placed into a 250 mL flask containing 100 mL of 1JN2 suspension with a concentration of 10^7^ CFU/mL for 2 days. After incubation, the roots were washed with sterile distilled water thrice and cut into 0.5 cm pieces and then fixed in 2.5% glutaraldehyde for 2 h. The root pieces were washed thrice for 10 min in 25 mM sodium phosphate buffer, and then, they were serially dehydrated in 50, 70, 80, and 90% acetone solutions for 15 min and, finally, in 100% acetone solution for 90 min. During this process, the acetone was changed every 30 min. Cucumber root pieces were transferred into tertbutyl alcohol thrice to displace acetone for 30 min each time and then freeze-dried in a freeze dryer (Thermo Scientific, Waltham, MA, USA). Finally, the root samples were observed by scanning electron microscopy (SU8010 SEM, Hitachi, Japan).

### 2.4 Collection and analysis of root secretion of cucumber plants

Cucumber root exudates were collected as followed: cucumber seedlings with four leaves were isolated from the soil, the roots of the cucumber were gently washed with sterilized water three times, and then transferred into Hoagland’s solution for 3 days. Then roots were washed again, and then, each root was divided into two parts, and each part was immersed into a 50 mL centrifuge tube containing 40 mL of sterilized water. Overall, 5 mL of *B. subtilis* 1JN2 (1*10^7^ CFU/mL) was added to tube A and designated as the treated group, and 5 mL of sterilized water was added to tube B and designated as the induced group. Cucumber plants without root separation were set as the control group. Each treatment contained 10 cucumber plants, and 3 independent repeats were conducted in this procedure. After 3 days, the solutions from tubes A and B in each treatment were collected together and filtered with 30-μm filter paper, then, the filtrates were freeze-dried.

For component analysis, 50 mg powder of each sample was transferred to 5 mL of EP tube and extracted with 3,000 μL of 75% methanol with 1% acetic acid. After vortexing for 30 s, the samples were homogenized at 40 Hz for 4 min and sonicated for 30 min in an ice-water bath. All the samples were centrifuged at 12,000 rpm (RCF = 13,800 (×*g*), *R* = 8.6 cm) for 15 min at 4°C. In total, 2,500 μL of the supernatant was dried under gentle nitrogen flow. The residues of each sample were reconstituted in 250 μL of 50% methanol with 0.1% formic acid containing internal standard. The samples were vortexed for 30 s and ultra-sonicated for 15 min in an ice bath. Then, all the samples were centrifuged at 12,000 rpm (RCF = 13,800 (×*g*), *R* = 8.6 cm) for 15 min at 4°C. The resulting supernatants were filtered through the 0.22-μm filter membrane. Then, they were transferred to 2 mL glass vials and stored at −80°C until the UHPLC–MS/MS analysis. The quality control (QC) sample was prepared by mixing an equal aliquot of the supernatants from all of the samples.

### 2.5 Analysis of rhizo-microbial diversity in cucumber plants treated with *Bacillus subtilis* 1JN2 and the exogenous combinations of amino acid

Two rounds of field experiment were conducted from September to November 2022 and 2023 in the vegetable experimental base of Huai’an Agricultural Technology Extension Center (E119.020292，N33.484059) to determine the diversity of the rhizo-microorganisms in cucumbers treated with *B. subtilis* 1JN2 and the exogenous combinations of amino acid induced from cucumber roots. The same variety of cucumber (Jindian 303) was used in the experiments. In the first round, suspension of *B. subtilis* 1JN2 was adjusted to 10^7^ CFU/mL and irrigated to 20 mL for each plant after transplantation of the cucumber plants. Three sites, which were placed 5 m apart, were selected for irrigation and each site consist of three cucumber plants. Rhizosphere soil samples were collected 1 day before treatment and 1 day, 3 days, 7 days, and 14 days after treatment. All the soil samples at five time points were stored at −80°C before sequencing and further carried out microbiota analysis by Majorbio Bio-Pharm Technology Co. Ltd. (Shanghai, China).

A second round of experiment was conducted at the same site on 2023, the experimental materials and methods were described above, but the strain treatment was replaced with an exogenous amino acid solution treatment. According to the results of cucumber root exudate analysis, four amino acids that were induced by the biocontrol strain were selected. Amino acid solutions in water were prepared such that they contained each of the selected compounds in equal amounts (2.5 mM glutamine, 2.5 mM tryptophan, 2.5 mM glycine, and 2.5 mM glutamate) and at a final concentration of 10 mM. In total, 10 mL of the amino acid mixture was irrigated to each plant, and the soil samples were collected at the same time as described above.

Total microbial genomic DNA was extracted from soil samples using the E.Z.N.A.^®^ Soil DNA Kit (Omega Bio-tek, Norcross, GA, USA), according to the manufacturer’s instructions. The quality and concentration of DNA were determined by 1.0% agarose gel electrophoresis and a NanoDrop2000 spectrophotometer (Thermo Scientific, United States) and kept at −80°C prior to further use. The hypervariable region V3-V4 of the bacterial 16S rRNA gene was amplified with primer pairs 338F (5′-ACT CCT ACG GGA GGC AGC AG-3′) and 806R (5′-GGA CTA CHV GGG TWT CTA AT-3′) (Liu et al., 2016) by a T100 Thermal Cycler PCR (BIO-RAD, USA). The PCR reaction mixture consists of 4 μL of 5 × Fast Pfu buffer, 2 μL of 2.5 mM dNTPs, 0.8 μL of each primer (5 μM), 0.4 μL of Fast Pfu polymerase, 10 ng of template DNA, and ddH2O to a final volume of 20 μL. PCR amplification cycling conditions were as follows: initial denaturation at 95°C for 3 min, followed by 27 cycles of denaturing at 95°C for 30 s, annealing at 55°C for 30 s, extension at 72°Cfor 45 s, single extension at 72°C for 10 min, and a final extension at 4°C. The PCR product was extracted from 2% agarose gel and purified using the PCR Clean-Up Kit (YuHua, Shanghai, China), according to the manufacturer’s instructions and quantified using Qubit 4.0 (Thermo Fisher Scientific, USA).

Purified amplicons were pooled in equimolar amounts and paired-end sequenced on an Illumina PE300 platform (Illumina, San Diego, USA), according to the standard protocols by Majorbio Bio-Pharm Technology Co. Ltd. (Shanghai, China). The raw sequencing reads were deposited into the NCBI Sequence Read Archive (SRA) database (Accession Number: PRJNA1019504).

Raw FASTQ files were de-multiplexed using an in-house perl script, quality-filtered by fastp version 0.19.6 (Chen et al., 2018), and then merged by FLASH version 1.2.7 (Magoc and Salzberg, 2011) with the following criteria: (i) the reads were truncated at any site receiving an average quality score of <20 over a 50-bp sliding window, and the truncated reads shorter than 50 bp were discarded; reads containing ambiguous characteristics were also discarded; (ii) only overlapping sequences longer than 10 bp were assembled according to their overlapped sequence. The maximum mismatch ratio of overlap region is 0.2, and reads that could not be assembled were discarded; and (iii) samples were distinguished according to the barcode and primers, and the sequence direction was adjusted. Then, the optimized sequences were clustered into operational taxonomic units (OTUs) using UPARSE 7.1 (Stackebrandt and Goebel, 1994; Edgar, 2013) with 97% sequence similarity level. The most abundant sequence for each OTU was selected as a representative sequence. The OTU table was manually filtered, i.e., chloroplast sequences in all samples were removed. To minimize the effects of sequencing depth on alpha and beta diversity measure, the number of 16S rRNA gene sequences from each sample was rarefied to 20,000, which still yielded an average Good’s coverage of 99.09%, respectively.

The taxonomy of each OTU representative sequence was analyzed by RDP Classifier version 2.2 (Wang et al., 2007) against the 16S rRNA gene database (e.g., Silva v138) using the confidence threshold of 0.7. The metagenomic function was predicted by Phylogenetic Investigation of Communities by Reconstruction of Unobserved States (PICRUSt2) (Douglas et al., 2020) based on OTU representative sequences. PICRUSt2 is software containing a series of tools as follows: HMMER was used to align OTU representative sequences with reference sequences. EPA-NG and Gappa were used to put OTU representative sequences into a reference tree. The castor was used to normalize the 16S gene copies. MinPath was used to predict gene family profiles and locate into the gene pathways. The entire analysis process was performed according to the protocols of PICRUSt2.

### 2.6 Statistical analysis

Statistical analysis was performed using a *t*-test, to compare the tested samples and control in the greenhouse experiment (SPSS 16.0). Statistical significance was determined at a *p*-value of <0.05.

Bioinformatic analysis of the soil microbiota was carried out using the Majorbio Cloud platform.^1^ Based on the OTU information, rarefaction curves and alpha diversity indices including observed OTUs, Chao1 richness, Shannon index, and Good’s coverage were calculated with Mothur v1.30.1 (Schloss et al., 2009). The similarity among the microbial communities in different samples was determined by principal coordinate analysis (PCoA) based on Bray–Curtis dissimilarity using the Vegan v2.5–3 package. The PERMANOVA test was used to assess the percentage of variation explained by the treatment along with its statistical significance using the Vegan v2.5–3 package. The linear discriminant analysis (LDA) effect size (LEfSe) (Segata et al., 2011)^2^ was performed to identify the significantly abundant taxa (phylum to genera) of bacteria among the different groups (LDA score > 2, *p* < 0.05). Since there is a multicollinearity problem among the 12 soil physicochemical properties/clinical parameters, the variance inflation factor (VIF) for each variable was estimated using the VIF function in the car package.^3^ The distance-based redundancy analysis (db-RDA) was performed using the Vegan v2.5-3 package to investigate the effect of soil physicochemical properties/clinical parameters on soil/gut bacterial community structure. Forward selection was based on Monte Carlo permutation tests (permutations = 9,999). Values of the *x*-axis and *y*-axis and the length of the corresponding arrows represented the importance of each soil physicochemical properties/clinical parameters in explaining the distribution of taxa across communities. Linear regression analysis was applied to determine the association between major physicochemical properties/clinical parameters identified by db-RDA analysis and microbial alpha diversity indices. The co-occurrence networks were constructed to explore the internal community relationships between the samples (Barberan et al., 2012). A correlation between two nodes was considered to be statistically robust if Spearman’s correlation coefficient was over 0.6 or less than −0.6 and the *p*-value was less than 0.01.

For the composition analysis of the root exudates of cucumber, the SIMCA16.0.2 software package (Sartorius Stedim Data Analytics AB, Umea, Sweden) was used for multivariate analysis. Data were scaled and logarithmically transformed to minimize the impact of both noise and high variance of the variables. After the transformation, principal component analysis (PCA), an unsupervised analysis that reduces the dimension of the data, was carried out to visualize the distribution and the grouping of the samples. Overall, 95% confidence interval in the PCA score plot was used as the threshold to identify potential outliers in the dataset.

To visualize group separation and find significantly changed metabolites, supervised orthogonal projections to latent structures-discriminate analysis (OPLS-DA) was carried out. Then, a 7-fold cross validation was performed to calculate the value of R2 and Q2. R2 indicates how well the variation of a variable is explained, and Q2 means how well a variable could be predicted. To check the robustness and predictive ability of the OPLS-DA model, permutation was further conducted 200 times. Afterward, the R2 and Q2 intercept values were obtained. In this study, the intercept value of Q2 indicates the robustness of the model, the risk of overfitting, and the reliability of the model, with smaller values indicating better performance.

Furthermore, the value of variable importance in the projection (VIP) of the first principal component in OPLS-DA analysis was obtained. It summarizes the contribution of each variable to the model. The metabolites with VIP > 1 and *p* < 0.05 (Student’s *t*-test) were considered as significantly changed metabolites. In addition, commercial databases including KEGG^4^ and MetaboAnalyst^5^ were used for pathway enrichment analysis.

## 3 Results

### 3.1 Biocontrol efficacy and colonization activity of *Bacillus subtilis* 1JN2 toward *Fusarium* wilt of cucumber

**Figure 1:**
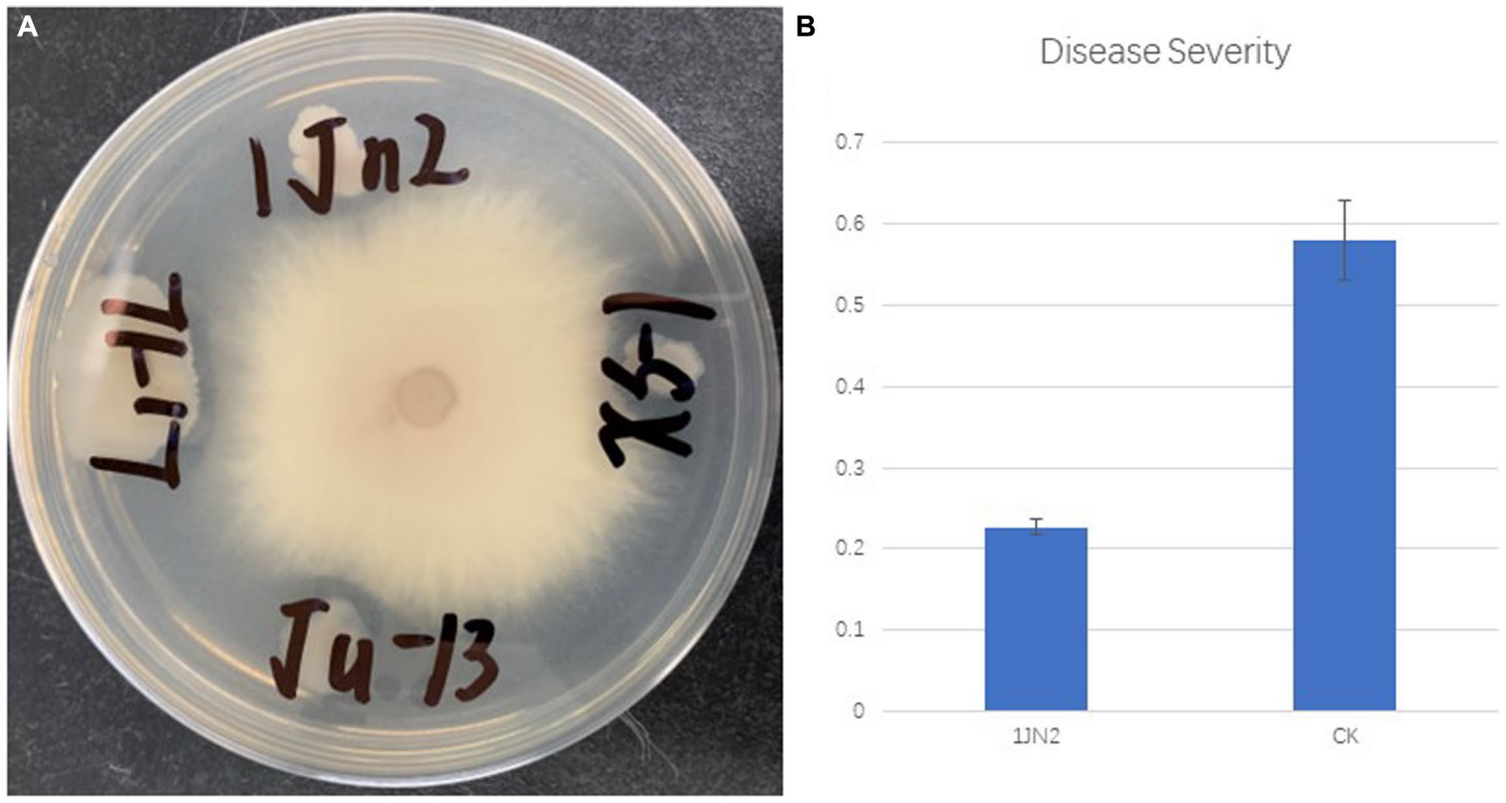
Plate antagonistic activity **(A)** and greenhouse control effect **(B)** of biocontrol strain 1JN2 against cucumber *Fusarium* wilt.

*Bacillus subtilis* isolate 1JN2 was proven to be an efficient biocontrol agent against *Ralstonia* wilt; the strain was isolated from the stem of a healthy ginger plant, where the *Ralstonia* wilt was spread severely (Yang et al., 2012). In this study, the plate antagonistic activity of the strain 1JN2 was tested first against *Fusarium oxyporum* which caused serious wilt on cucumber. According to the results, the strain can inhibit the growth of *F. oxyporum,* but the inhibition effect was not significant. The antagonistic radius reached 1–3 mm on LB plate (Figure 1A). The biocontrol efficacy against *Fusarium* wilt of the strain in greenhouse experiment was detected. Interestingly, compared with the plate antagonistic activity, the strain significantly reduced the occurrence of cucumber *Fusarium* wilt in greenhouse experiment. Compared with the blank control group, the strain could significantly reduce the occurrence of *Fusarium* wilt disease in artificially controlled greenhouse conditions (Figure 1B). The disease severity of the blank control group reached 58%, while after the pretreatment of biocontrol bacteria, the disease severity of the treatment group was only 22.7%. All the results above indicated that the strain could effectively decrease cucumber *Fusarium* wilt indirectly.

### 3.2 The biocontrol isolate 1JN2 can effectively colonize on the root of cucumber

**Figure 2:**
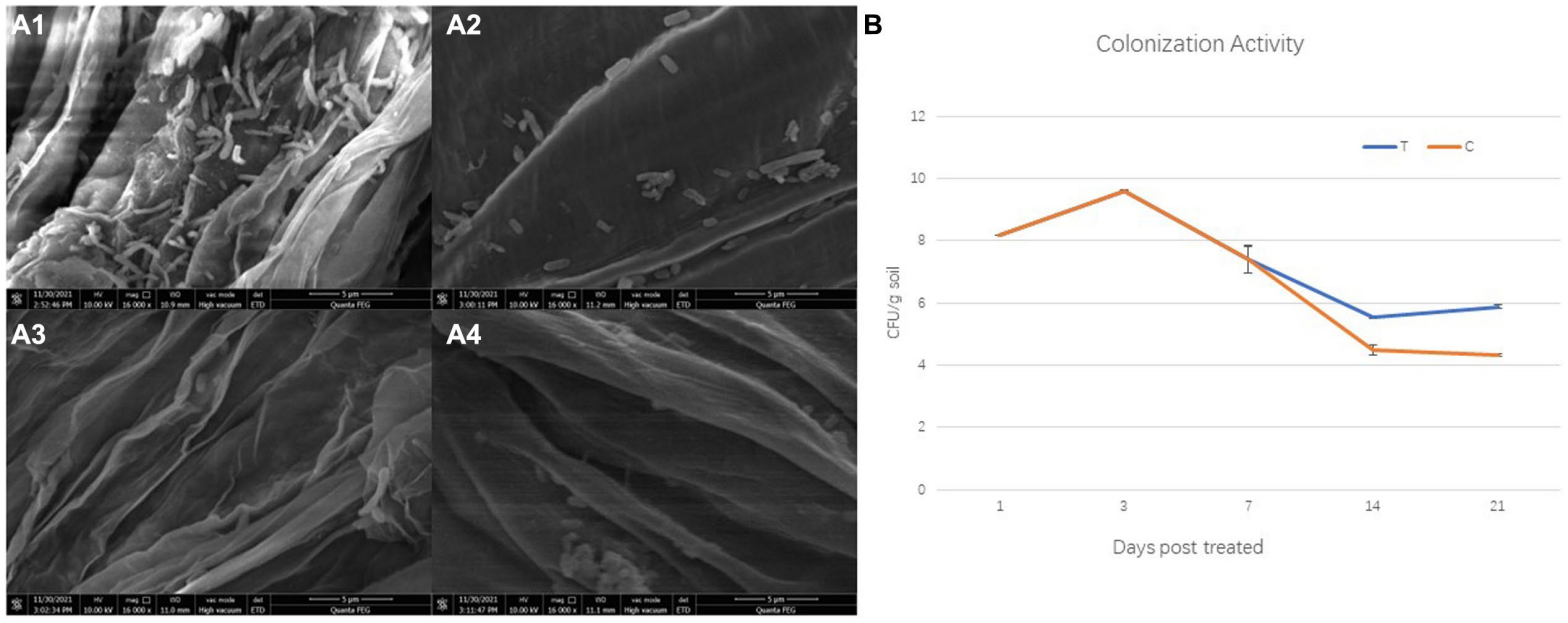
Colonization of the biocontrol strain 1JN2 on cucumber roots. **(A1,A2)** Were root tip and taproot of cucumber treated with 1JN2 suspension, **(A3,A4)** were root tip and taproot of cucumber treated with water. **(B)** Shows the survival of 1JN2 in the soil with (T) or without cucumber (C).

Colonization ability is generally considered as the first but crucial step to achieve the effect of biocontrol strains. Whether direct or indirect actions, biocontrol strains must accumulate enough population density to achieve disease control or microecological reset. Moreover, the colonization activity of the isolate 1JN2 on cucumber root was detected through plate spreading and SEM analysis. First, the survival of the strain in the presence or absence of cucumber plants was detected. Cucumber plants are promotive for the survival of the strain 1JN2 in soil. The population density of the strain 1JN2 in soil increased slightly from 1 to 3 days after inoculation but decreased significantly from the third day. The decline trend of the control group without cucumber plants was more obvious, which was only 10^4^ CFU/g soil on the 14th day after inoculation. In contrast, the density of the strain in the cucumber plant treatment group was close to 10^6^ CFU /g soil on the 14th day after inoculation and began to rise slowly and reached 10^6^ CFU/g soil 21 days after inoculation (Figure 2B). The colonization sites of the strain on cucumber root were detected by SEM. Compared with the taproot, the strain preferred to colonize on the root tip of cucumber (Figure 2A). It may be because the root tip provides more nutrition and colonization sites.

### 3.3 Root exudate profiles of cucumber plants treated with *Bacillus subtilis* 1JN2

First, non-target detection for the root exudates from cucumber was performed with LC–MS/MS in order to identify the types of components that changed in cucumber root exudates after treatment with the biocontrol strain 1JN2 (Supplementary Figure S1). Compared with the blank control, the main components of cucumber root exudates in the biocontrol bacteria treatment group included lipids and lipid-like molecules (30.79%), organoheterocyclic compounds (9.49%), phenylpropanoids and polyketides (9.49%), benzenoids (6.94%), organic oxygen compounds (5.32%), and organic acids and derivatives (4.4%), while in the biocontrol bacterial-induced group, similar results were obtained, except for that the content of organic acids and derivatives (6.59%) was higher than that of organic oxygen compounds (5.27%). When we focus on the differences between the 1JN2-treated group and induced group, we found that sterebin G, choline, ginsenoside Rh1, and sulfoquinovosyl diacylglycerol were higher in the 1JN2-treated group, and S−adenosylmethioninamine, agnuside, glycine, and triethanolamine were higher in the 1JN2-induced group. From the above results, it can be inferred that alkaloids and their derivatives are the representative components of the treatment group of the biocontrol strain, which may come from the metabolites of the biocontrol strain 1JN2. The amino acids in the induction group may come from the changes in cucumber root exudates after the treatment of biocontrol strain. Because the strain 1JN2 showed strong control effect on *Fusarium* wilt in greenhouse experiment, the direct inhibition effect on *Fusarium* wilt pathogen in plate antagonistic experiment was not significant. Moreover, we speculated that the strain could control *Fusarium* wilt mainly by an indirect method, changing the microecology of rhizosphere. To identify the specific components that may play a role in the microecological reset, we analyzed the amino acid composition of cucumber root exudates treated with the biocontrol strain 1JN2.

**Figure 3:**
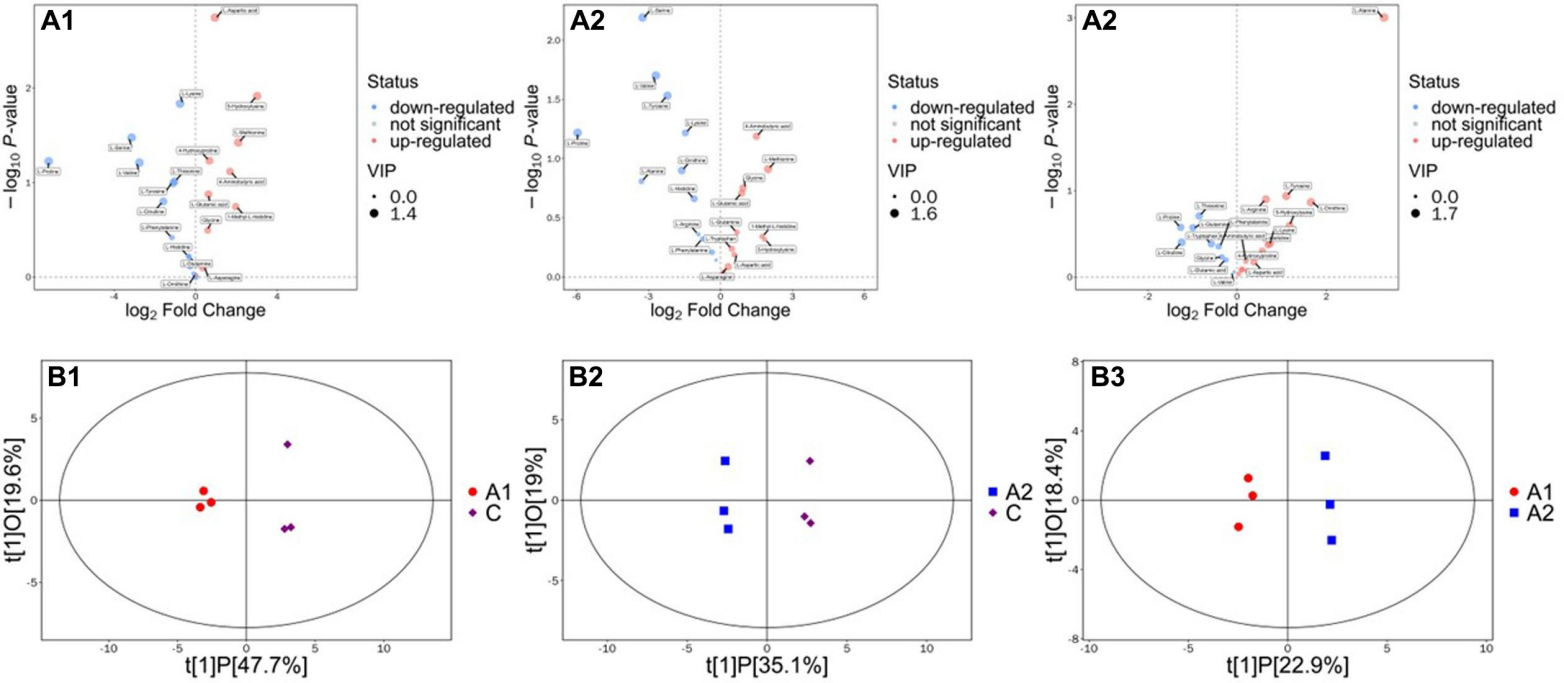
Analysis on the differences of amino acids between the strain treatment group and strain induction group. **(A1–A3)** Mean the differences of amino acids between the strain treatment group to CK, strain induction group to CK, and strain treatment group and strain induction group. **(B1–B3)** mean the score scatter plot of the OPLS-DA model for the strain treatment group to CK, strain induction group to CK, and strain treatment group and strain induction group.

According to the results, each group has its own unique amino acids compared with the control group. In the biocontrol strain-inoculated group (group A1), 5-hydroxylysine, L-methionine, 1-methyl-L-histidine, 4-aminobutyric acid, and L-aspartic acid are the top five amino acids that significantly upregulated compared with the blank control. While in the biocontrol strain-induced group (group A2), four out of the top five upregulated amino acids are the same as the strain inoculated group include 5-hydroxylysine, L-methionine, 1-methyl-L-histidine, and 4-aminobutyric acid. The only amino acid different from the A1 group is glycine rather than L-aspartic acid. When we focus on the differences between the A1 and A2 groups, the content of glutamine, L-tryptophan, glycine, and L-glutamic acid in A2 was significantly higher than those in the blank control group and also higher than those in the A1 group (Figure 3). Therefore, this group of amino acids is considered to be induced by biocontrol strain in cucumber roots and may play a key role in the assembly of cucumber rhizosphere microecology.

### 3.4 Recruitment of rhizosphere bacterial communities of cucumber plants treated by biocontrol strain and mixture of amino acids

**Figure 4:**
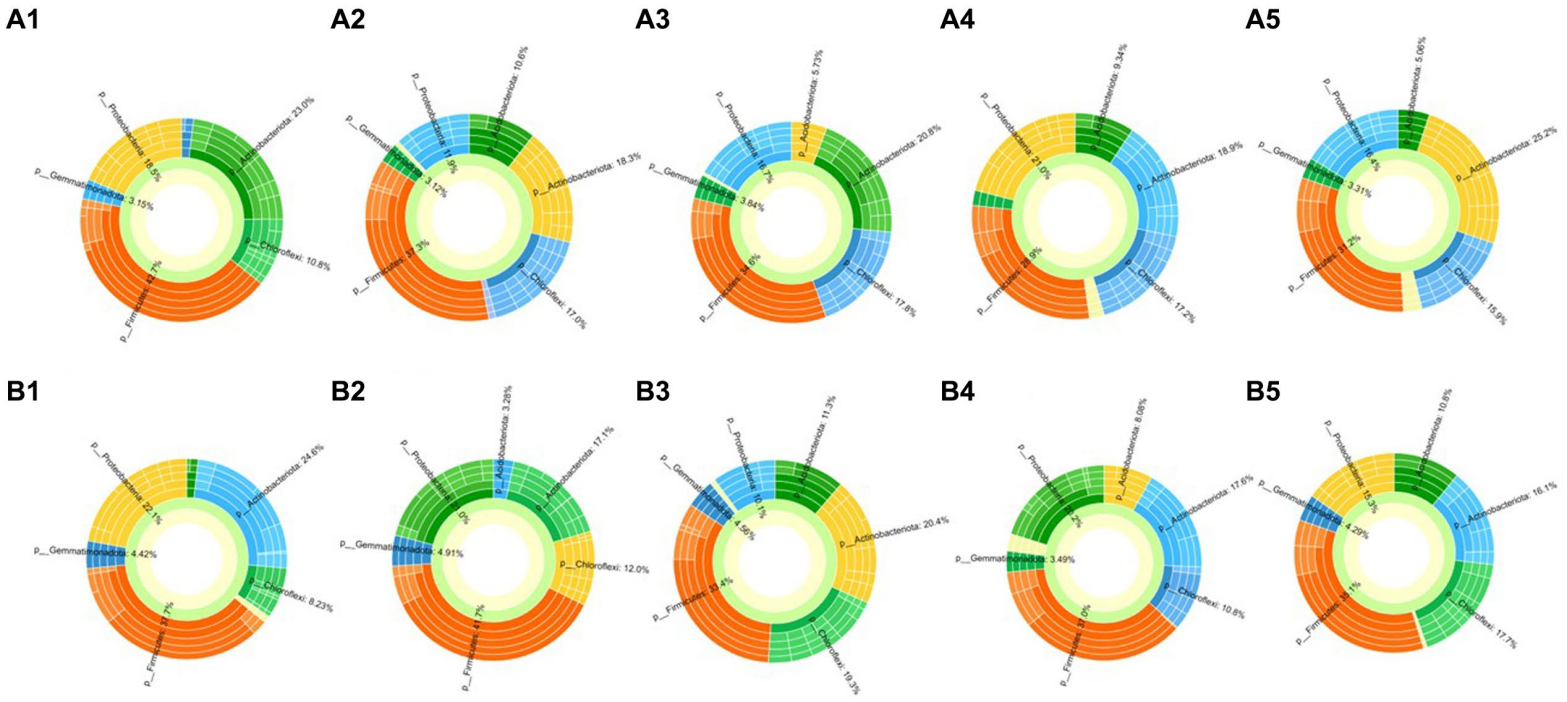
Community analysis of rhizosphere bacteria of cucumber (sunburst plot on the genus level). **(A1–A5)** Means soil samples collected 1 day before treated with 1JN2 suspension and 1 day, 3 days, 7 days, and 14 days after treatment. **(B1–B5)** Means soil samples collected 1 day before treated with amino acid solution and 1 day, 3 days, 7 days, and 14 days after treatment.

Bacterial communities in the rhizosphere of cucumber treated by biocontrol strain and mixture of amino acids were characterized with Illumina HiSeq sequencing. In total, 1,537,449 high-quality sequences were obtained, and each sample contained 27,341 to 90,019 sequences. All the samples were analyzed by PLS-DA on the genus level first, regardless of the biocontrol bacteria treatment group or the amino acids combination treatment group, and it was observed that the samples at different time points before and after treatment were significantly different (Supplementary Figure S2), which means that treatments with biocontrol strain or mixture of amino acids significantly changed the structure of rhizobacterial communities of cucumber. On the phylum level, whether in the treatment group or the control group, the dominant populations of cucumber rhizosphere bacteria are mainly concentrated in six phyla, namely, *Firmicutes*, *Actinobacteriota, Proteobacteria, Chloroflexi, Gemmatimonadota,* and *Acidobacteriota* (Figure 4). Among them, the bacterial population density of *Firmicutes* in soil samples at the last four time points in the biocontrol bacteria treatment group decreased compared with that before treatment. However, there was no significant change in *Firmicutes* bacteria in the amino acid mixture treatment group. The bacterial populations of *Actinobacteriota, Proteobacteria, Chloroflexi*, and *Gemmatimonadota* had no significant changes in either the biocontrol bacteria treatment group or the amino acid mixture treatment group. The most significant change occurred in the bacterial population of *Acidobacteriota*. After the treatment of biocontrol strain or amino acid mixture, the bacterial population of *Acidobacteriota* showed a significant upward trend, that is, from 1.88 and 1.3% before treatment to 11% after treatment (Supplementary Figure S3).

**Figure 5:**
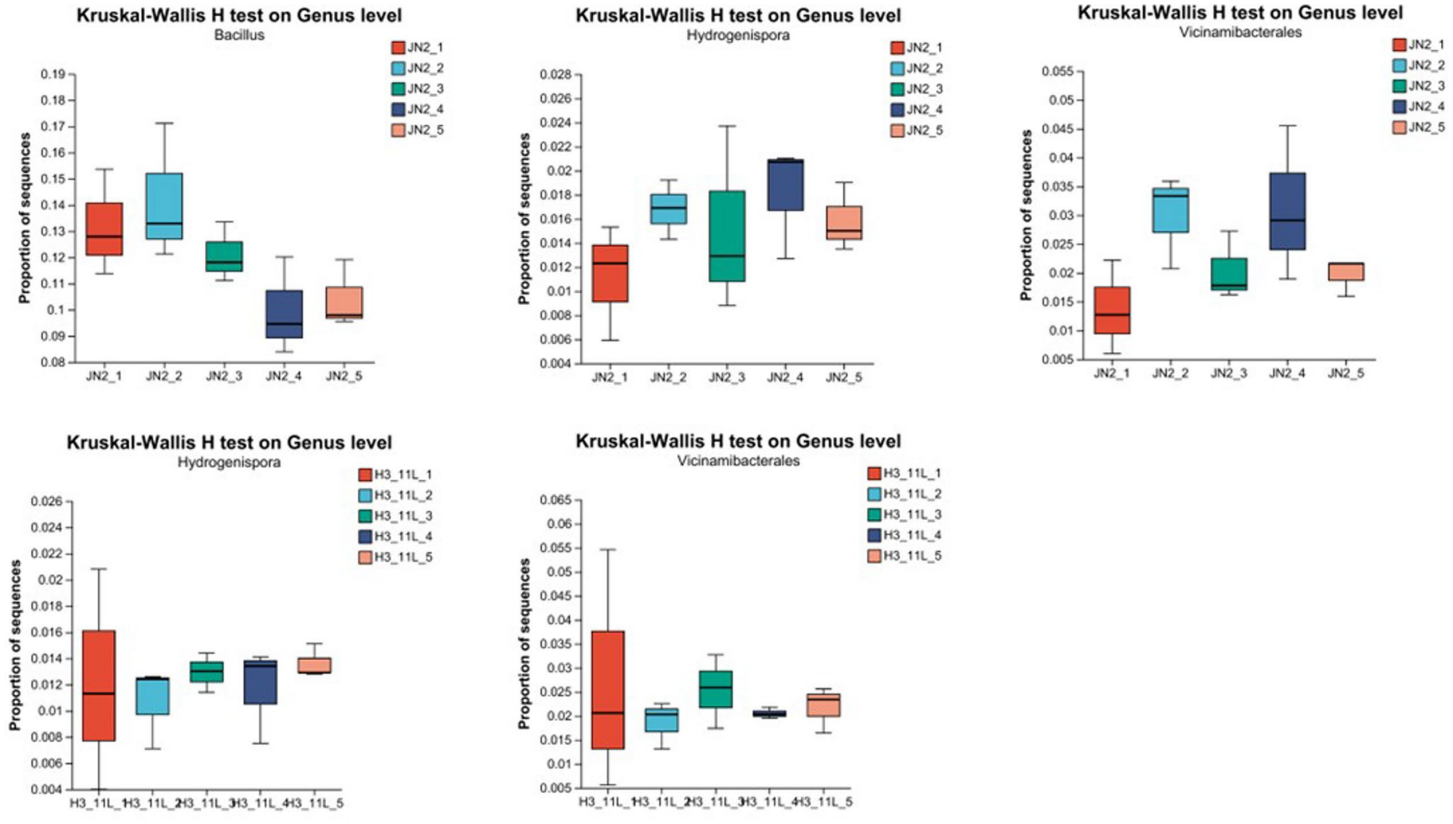
Analysis of single species difference test (Kruskal–Wallis H test on the genus level). 1JN2_1 to 1JN2_5 means soil samples collected 1 day before treated with 1JN2 suspension and 1 day, 3 days, 7 days, and 14 days after treatment. H3_11L_1 to H3_11L_5 means soil samples collected 1 day before treated with amino acid solution and 1 day, 3 days, 7 days, and 14 days after treatment.

Focusing on the genus level, although the total number of bacteria from *Firmicutes* in the biocontrol strain treatment group was decreasing, the bacterial diversity of *Firmicutes* was increasing, and among them, bacteria of the genus *Hydrogenispora* increased significantly after treatment. The same trend also appeared in the amino acid mixture treatment group. Although there was no significant change in the number of bacteria of *Firmicutes* in the treatment group, bacteria belong to the genus *Hydrogenispora* increased significantly after treatment. Among the *Acidobacteria* bacteria with the largest changes in the biocontrol strain treatment group and the amino acid mixture treatment group, bacteria of the genus *Vicinamibacterales* were the dominant population. Among the phyla *Actinobacteria, Proteobacteria, Chloroflexi*, and *Gemmatimonadota* without obvious changes, the dominant populations mainly include the genera of *Marmoricola, Nocardioides, Streptomyces, Sphingomonas*, and *Gemmatimonadaceae* (Supplementary Figure S4). The number of *Bacillus* was the largest in all dominant populations, exceeding 20% in all treatment groups (Figure 5).

Based on the above results, we can speculate that the bacteria of *Hydrogenispora* and Vicinamibacteriales may play a key role in the process of the biocontrol strain 1JN2, resetting the bacterial community structure of cucumber rhizosphere, which may come from the recruitment of significantly increased amino acids in cucumber root exudates.

## 4 Discussion

Using beneficial microorganisms in the environment to control crop diseases has a good application prospect in agricultural sustainable production (Mendes et al., 2013). More and more biocontrol strains began to be applied in actual agricultural production after laboratory screening (Luo et al., 2022). However, many biocontrol strains that have shown significant efficacy in the laboratory have been eliminated due to the unstable effect of disease control on the field. Therefore, elucidating the action mechanism of biocontrol strains after application can provide theoretical guidance for the practical application of biocontrol strains. In the current screening process of biocontrol strains, the direct inhibition of pathogenic microorganisms is often considered as an important indicator with biocontrol potential (Yang et al., 2012). However, some biocontrol bacteria with a significant antagonistic effect of plate have no obvious effects on disease control in greenhouse experiments or field experiments. On the contrary, some strains without or with little plate antagonistic activity showed significant disease control effect after treating crops (Zheng et al., 2018). Either of the above situations may be related to the establishment on the community ability of biocontrol strains after application or the ability to reset the microecology.

The biocontrol strains with significant plate antagonistic activity may not survive in the application environment due to their poor competitive ability, showing no biocontrol effect. The strains without plate antagonistic activity may become the dominant population in the environment due to their strong colonization ability and can reset the microecology, shaping a microecology that is beneficial to the host plant, to avoid pathogen infection (Bashan et al., 2014; Zmora et al., 2018). The biocontrol *Bacillus* 1JN2 applied in this study belongs to the latter. Although the antagonistic activity on the plate is not obvious, it has shown significant control effect on cucumber *Fusarium* wilt in the greenhouse experiment. This indirect biocontrol effect depends on the ecological fitness of biocontrol strains after application. This ecological effect includes both the direct effect of biocontrol bacteria and indigenous microorganisms and the indirect interaction between biocontrol bacteria, host plants, and indigenous microorganisms.

Among the indigenous microbial populations in the plant rhizosphere, there is a class of factors that are considered to be helper of pathogen infection. The inhibition of these microorganisms can also prevent pathogen infection (Li et al., 2022). Wen et al. (2020) analyzed the shift of rhizobacterial structure between resistant and susceptible phenotypes of cucumber against *Fusarium* wilt and found that four organic acids (citric acid, pyruvate acid, succinic acid, and fumarate) were released at higher abundance by the Foc-susceptible cultivar compared with the resistant cultivar., which may be responsible for the recruitment of *Comamonadaceae*, a potential beneficial microbial group. This result indicates that the pathogen-resistant host plants attract the attention of beneficial microorganisms through the change in root exudates, which is also known as “cry for help” (Dicke and Baldwin, 2010). In this study, another mode of “cry for help,” mediated by a biocontrol agent, was proven. By inoculating biocontrol strain, the amino acids in cucumber root exudates were significantly increased, which attracted two groups of bacteria, *Hydrogenispora* and Vicinamibacteriales, to prevent the infection of *Fusarium* wilt. Amino acids involved in this process include glutamine, L-tryptophan, glycine, and L-glutamic acid.

Organic acids are the most important components of root exudates of host plants, which will change with the changes in host plant varieties, physiological periods, and other factors (Wang et al., 2023). Among them, amino acids are often used as the most common medium in the “cry for help” mechanism. Local infection of cucumber roots by *Fusarium oxysporum* f.sp. *cucumerinum* increased tryptophan but reduced raffinose exudation, and these changes enhanced root colonization by the beneficial bacterium *Bacillus amyloliquefaciens* SQR9 (Liu et al., 2017). Not only the pathogen infection but also the inoculation of biocontrol strains could change the amino acid content in the root exudates of host plants (Kuzmicheva et al., 2017). Inoculation with biocontrol agent AR156 upregulated metabolites in tomato root exudates, such as lactic acid and hexanoic acid, and induced the growth and motile ability of *in vitro B. cereus* AR156 cells. Exogenously applying hexanoic acid and lactic acid to tomato plants showed positive biocontrol efficacy (46.6 and 39.36%) against tomato bacterial wilt. Similar results were obtained in cucumber in this study, while the main upregulated amino acids include glutamine, L-tryptophan, glycine, and L-glutamic acid. In cucumber root exudates, amino acids comprise the most abundant source of reduced carbon (Liu et al., 2007). Chemotaxis toward root exudates is a well-appreciated trait of rhizosphere-colonizing bacteria, which are essential for root colonization (de Weert et al., 2002). Root exudates are known to induce a number of additional phenotypes in rhizosphere-dwelling bacteria, for example, promotion of polychlorinated biphenyl degradation (Toussaint et al., 2012) and induction of biofilm formation by *B. subtilis* on roots (Rudrappa et al., 2008). In this study, after the treatment of the biocontrol strain 1JN2 or exogenous amino acid mixture, *Bacillus* spp. of cucumber rhizosphere changed significantly, which infers that the amino acid root exudates induced by biocontrol bacteria can recruit beneficial microorganisms through chemotaxis.

When we focus on the rhizobacterial community in cucumbers treated with biocontrol strain 1JN2 and exogenous amino acid mixture, bacteria of *Hydrogenispora* and Vicinamibacteriales increased significantly. Among them, *Hydrogenispora* are reported to be anaerobic bacteria that are capable of decomposing diverse carbon sources under anoxic environments and have identified as the unique core taxa for rice soils (Jiao et al., 2019). Bacteria from the genus *Hydrogenispora* may be involved in denitrification and biodegradation of organic carbon, promoting nutrient cycling in rhizosphere soil of host plants (Wongkiew et al., 2022). Bacteria of the order Vicinamibacteriales were reported to be the main population in soils and peatlands, and they are especially abundant in acidic peat bogs. The genome of a bacterium of the class Vicinamibacteria contained a set of genes encoding bacterial microcompartments (metabolosomes) that have not previously been described in acidobacteria and are probably involved in the metabolism of L-rhamnose (Dedysh et al., 2022). Bacteria from the order Vicinamibacteriales involved in P solubilization was also proven by a genome-centric metagenomics approach (Wu et al., 2021). Based on the above, we can conclude that the biocontrol strain 1JN2 can induce the expression of four amino acids in cucumber root exudates. This change in root exudates reassembled cucumber rhizobacterial community through chemotaxis, increased the bacterial diversity, significantly increased the population density of *Hydrogenispora* and Vicinamibacteria, promoted the nutrient cycling of soil, and improved the resistance of cucumber against *Fusarium* wilt.

## Data availability statement

The datasets presented in this study can be found in online repositories. The names of the repository/repositories and accession number(s) can be found in the article/Supplementary material.

## Author contributions

WY: Supervision, Writing – review & editing. XL: Formal analysis, Writing – original draft. HY: Investigation, Writing – original draft. YS: Investigation, Methodology, Writing – original draft. DW: Formal analysis, Writing – original draft. YD: Software, Writing – original draft. YL: Funding acquisition, Supervision, Writing – review & editing.

## Footnotes

^1^ https://cloud.majorbio.com

^2^ http://huttenhower.sph.harvard.edu/LEfSe

^3^ https://cran.r-project.org/web/packages/car/car.pdf

^4^ http://www.genome.jp/kegg/

^5^ http://www.metaboanalyst.ca/
