# Supplementary figures and images for: Recruitment of beneficial cucumber rhizosphere microbes mediated by amino acid secretion induced by biocontrol Bacillus subtilis isolate 1JN2
Source: Front Microbiol. 2024 Apr 4;15:1379566. doi: 10.3389/fmicb.2024.1379566 (PMC11024430; doi:10.3389/fmicb.2024.1379566)

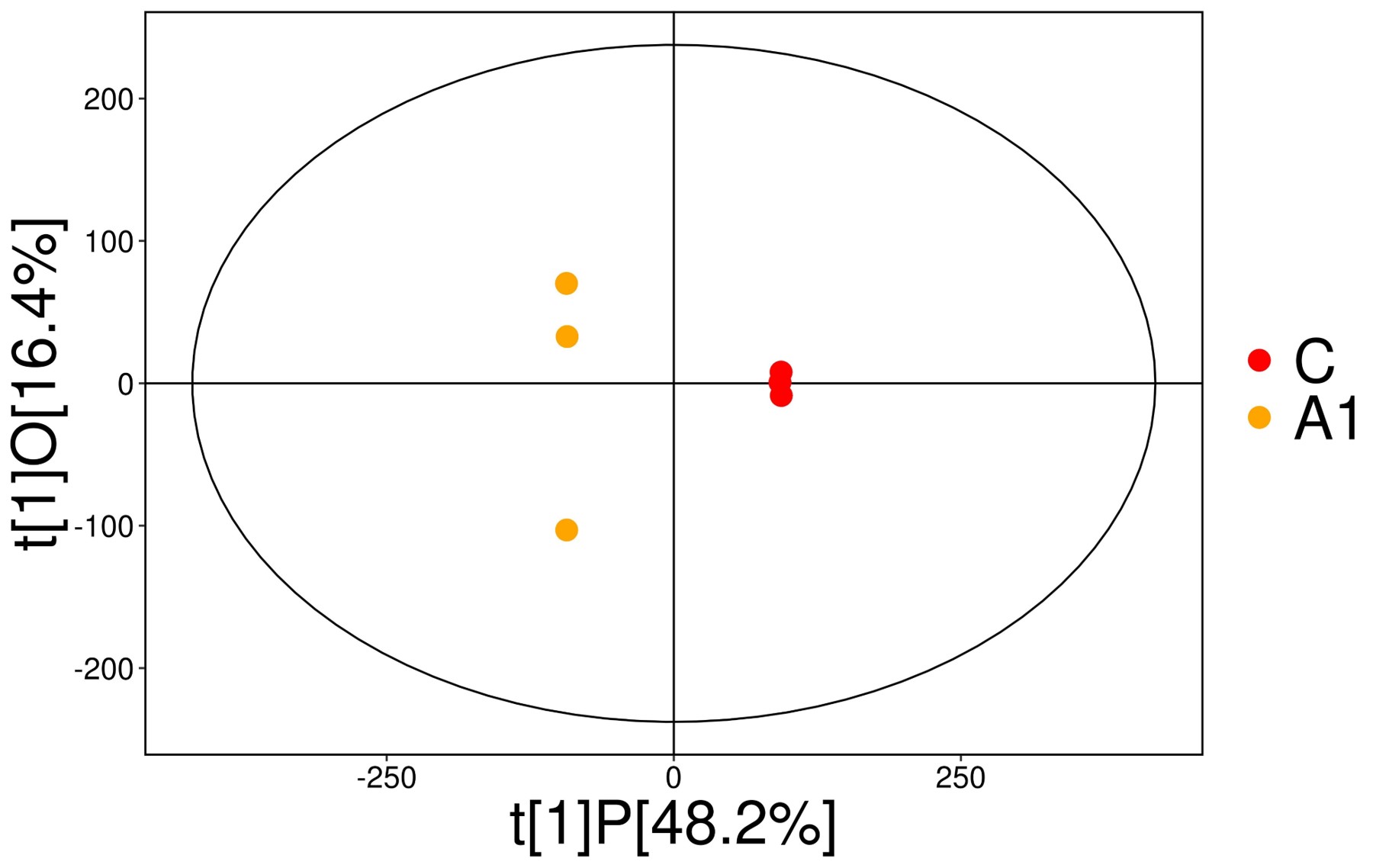

Supplement: Supplementary file 1 [file Data_Sheet_1.zip › Supplementary Figures/FigureS1-A1.jpg]

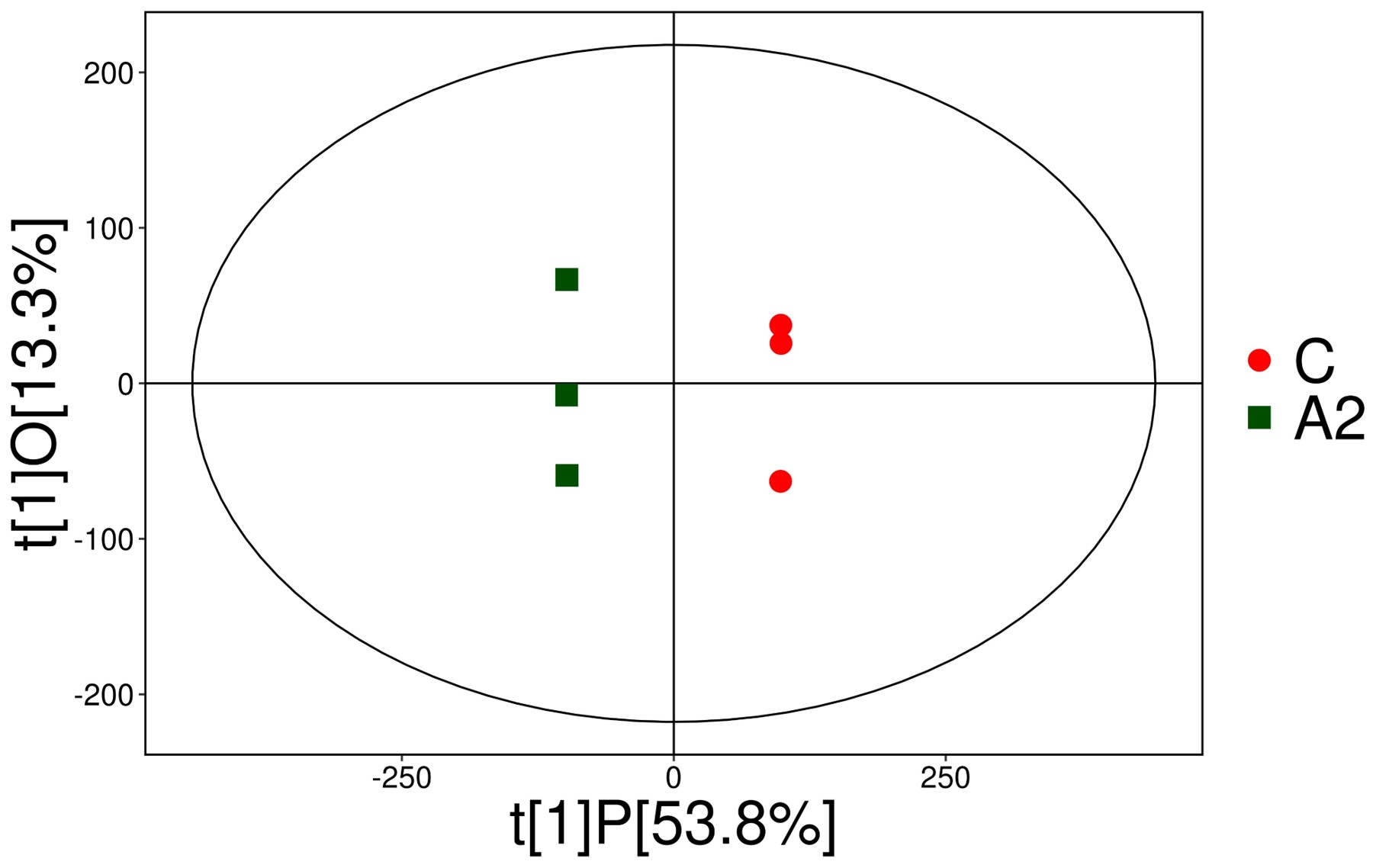

Supplement: Supplementary file 1 [file Data_Sheet_1.zip › Supplementary Figures/FigureS1-A2.jpg]

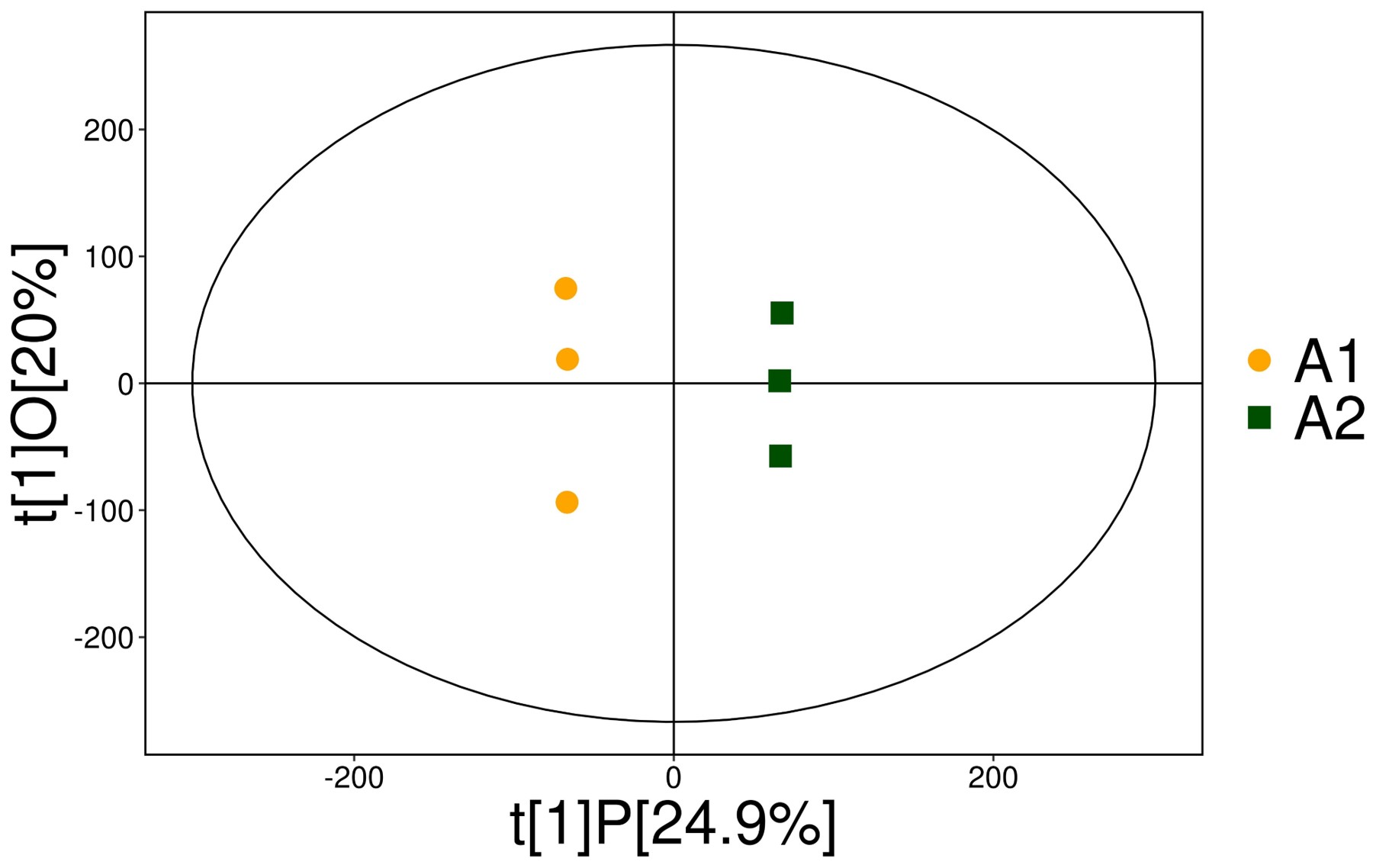

Supplement: Supplementary file 1 [file Data_Sheet_1.zip › Supplementary Figures/FigureS1-A3.jpg]

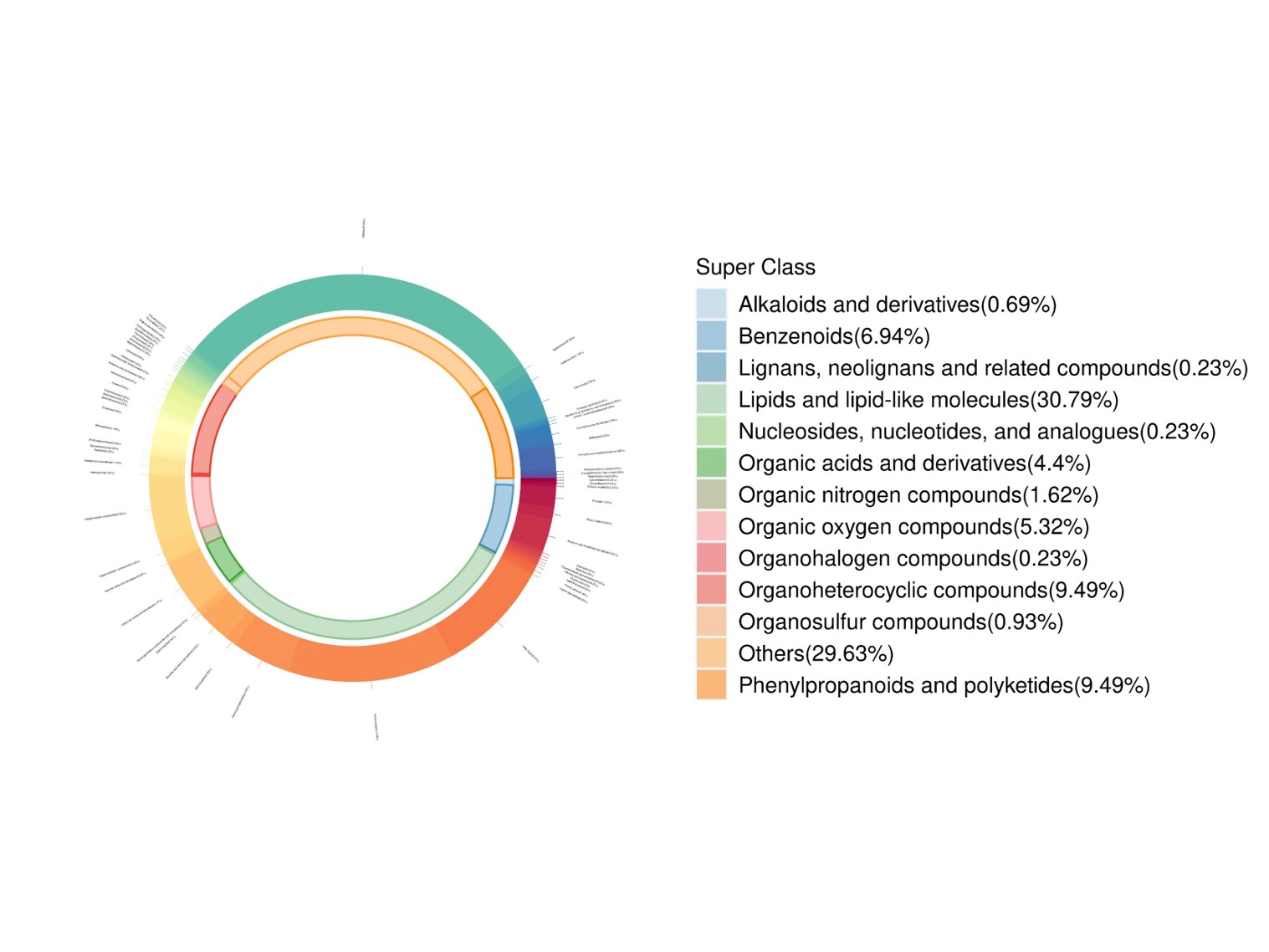

Supplement: Supplementary file 1 [file Data_Sheet_1.zip › Supplementary Figures/FigureS1-B1.jpg]

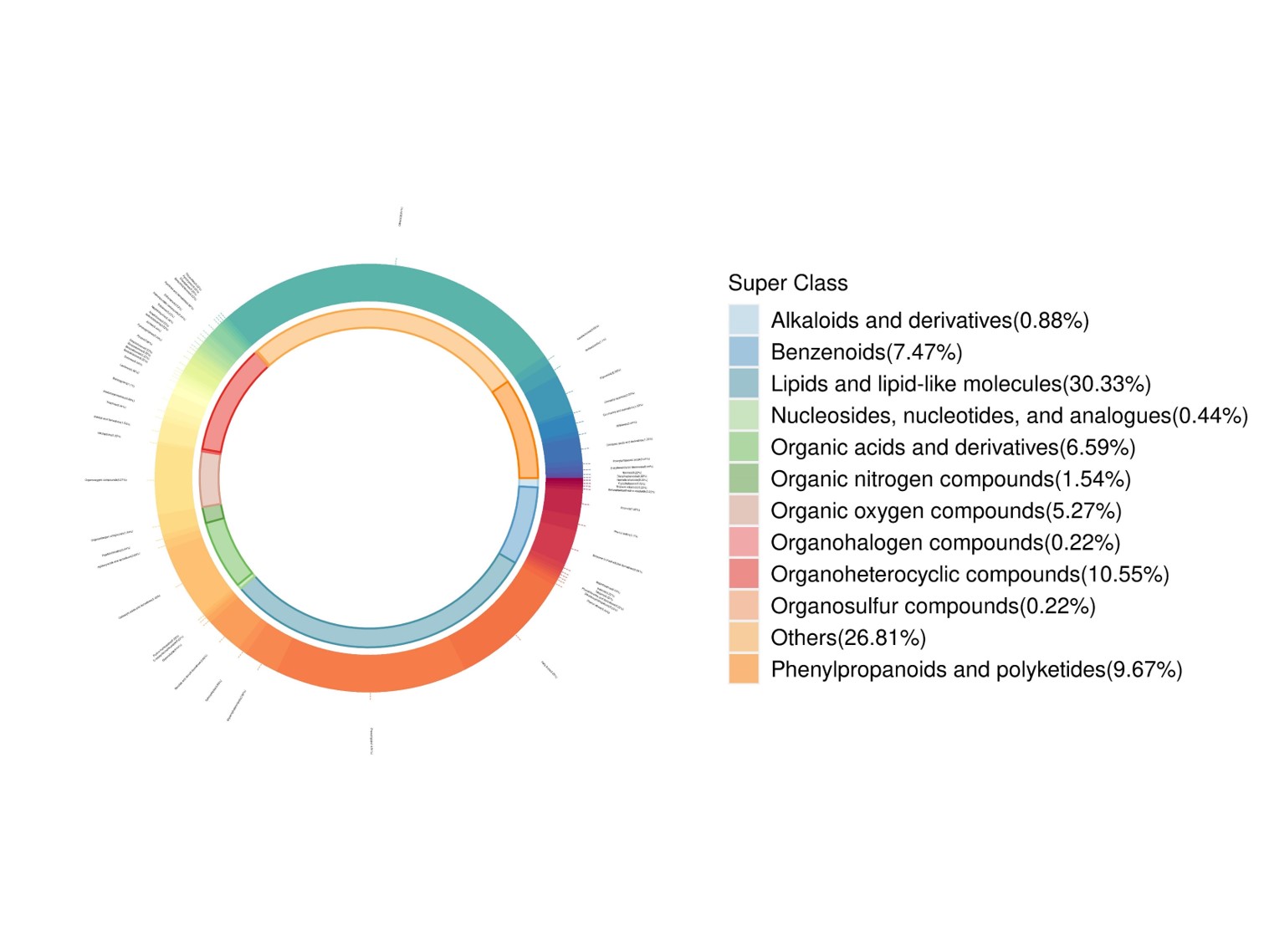

Supplement: Supplementary file 1 [file Data_Sheet_1.zip › Supplementary Figures/FigureS1-B2.jpg]

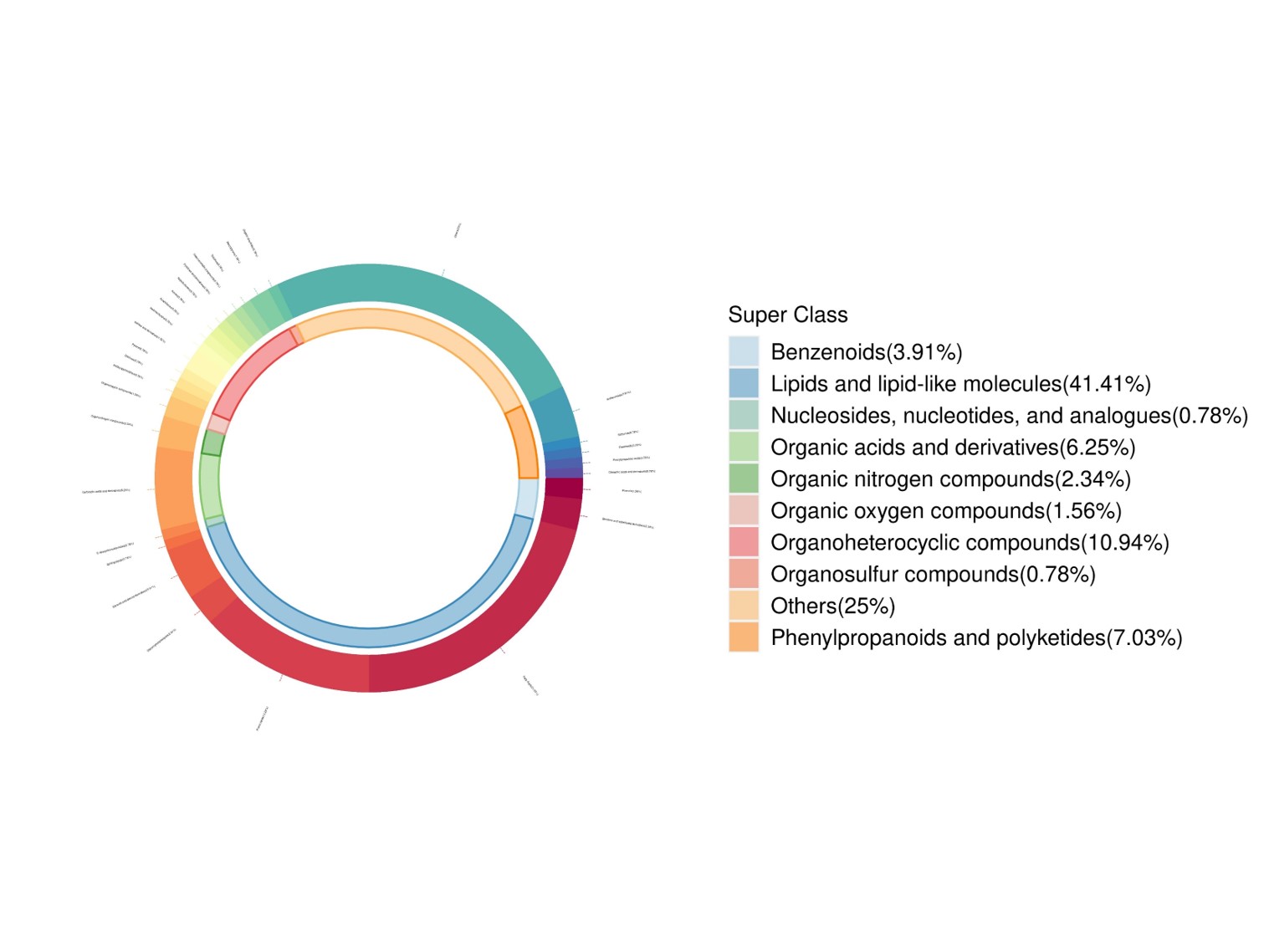

Supplement: Supplementary file 1 [file Data_Sheet_1.zip › Supplementary Figures/FigureS1-B3.jpg]

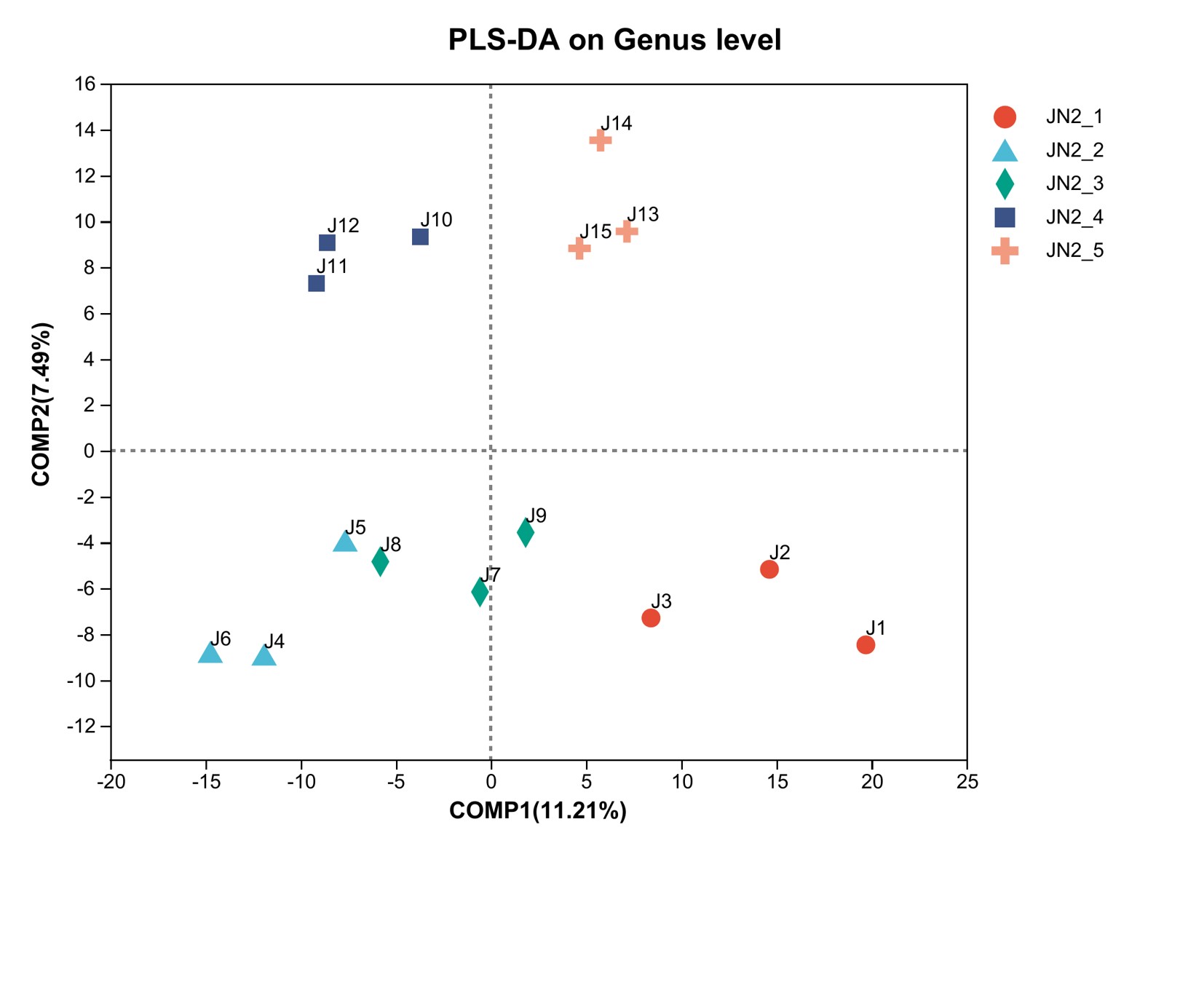

Supplement: Supplementary file 1 [file Data_Sheet_1.zip › Supplementary Figures/FigureS2-A.jpg]

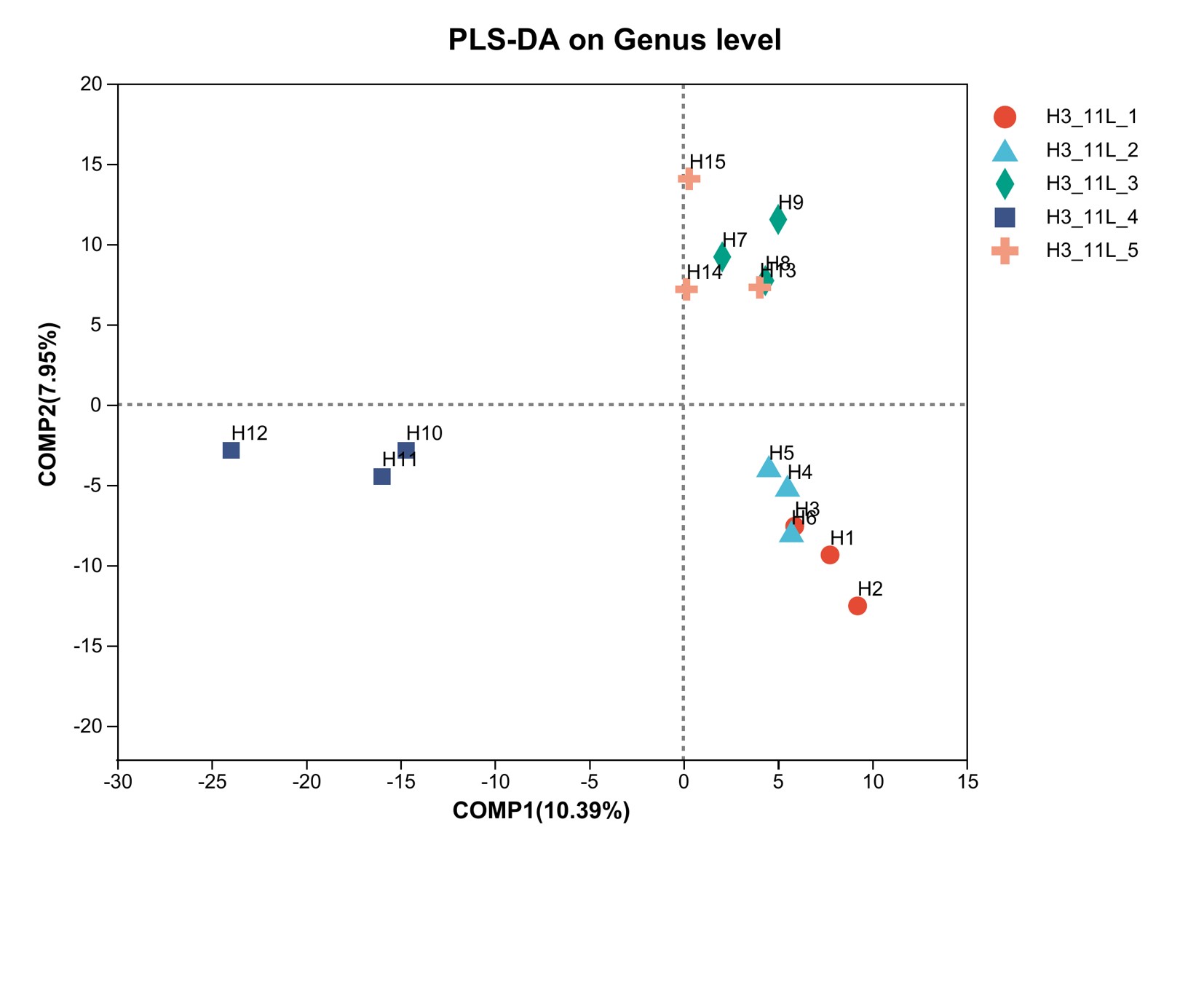

Supplement: Supplementary file 1 [file Data_Sheet_1.zip › Supplementary Figures/FigureS2-B.jpg]

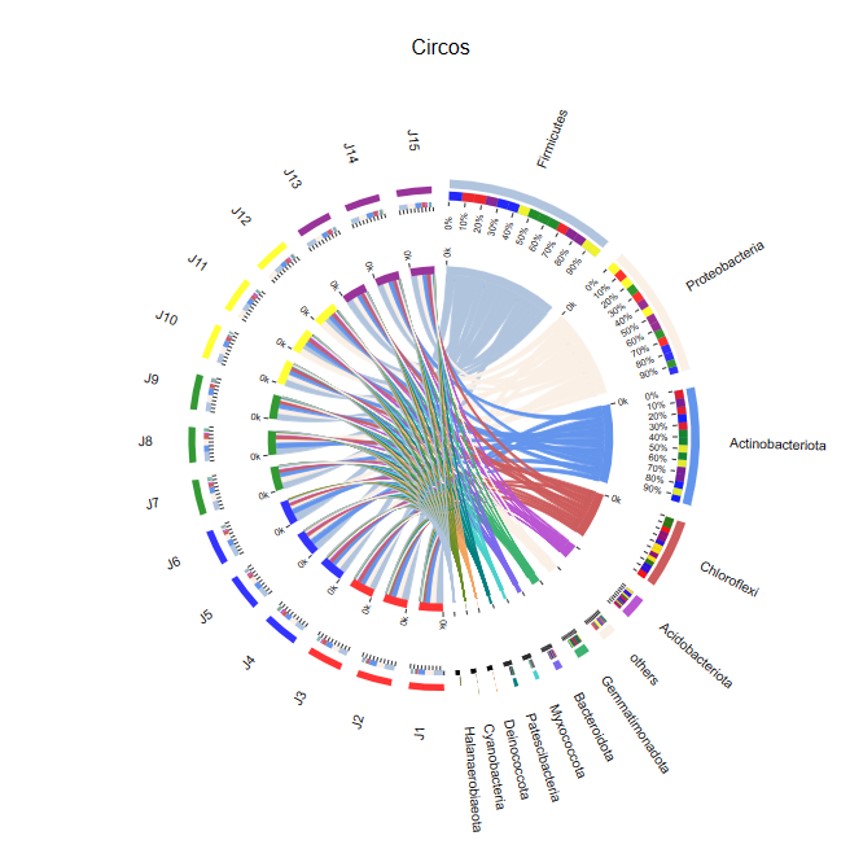

Supplement: Supplementary file 1 [file Data_Sheet_1.zip › Supplementary Figures/FigureS3-A.jpg]

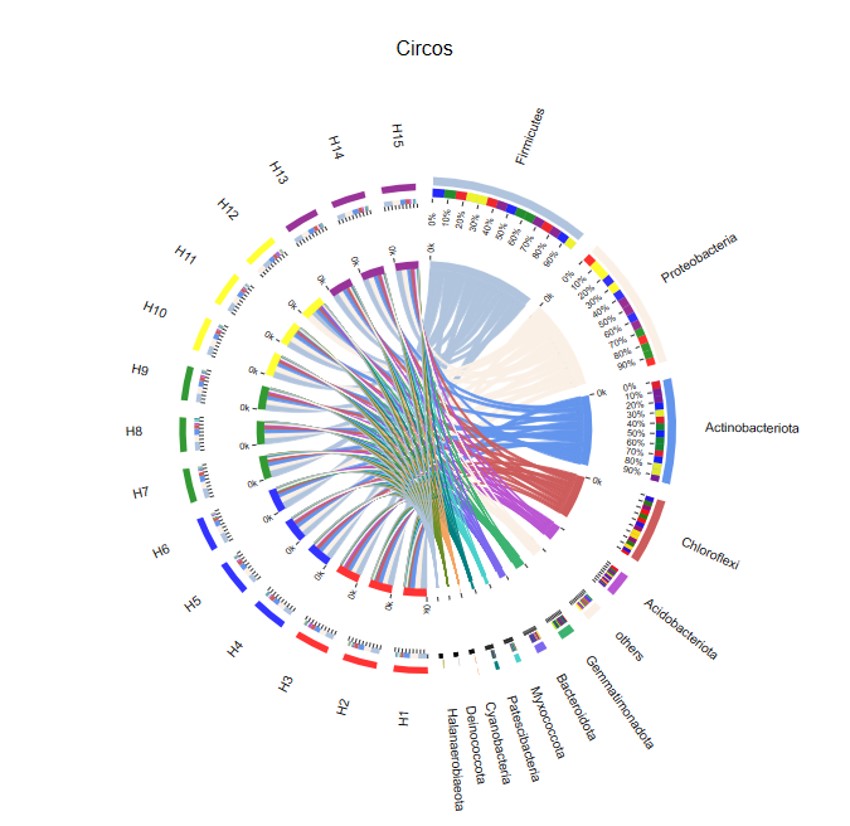

Supplement: Supplementary file 1 [file Data_Sheet_1.zip › Supplementary Figures/FigureS3-B.jpg]

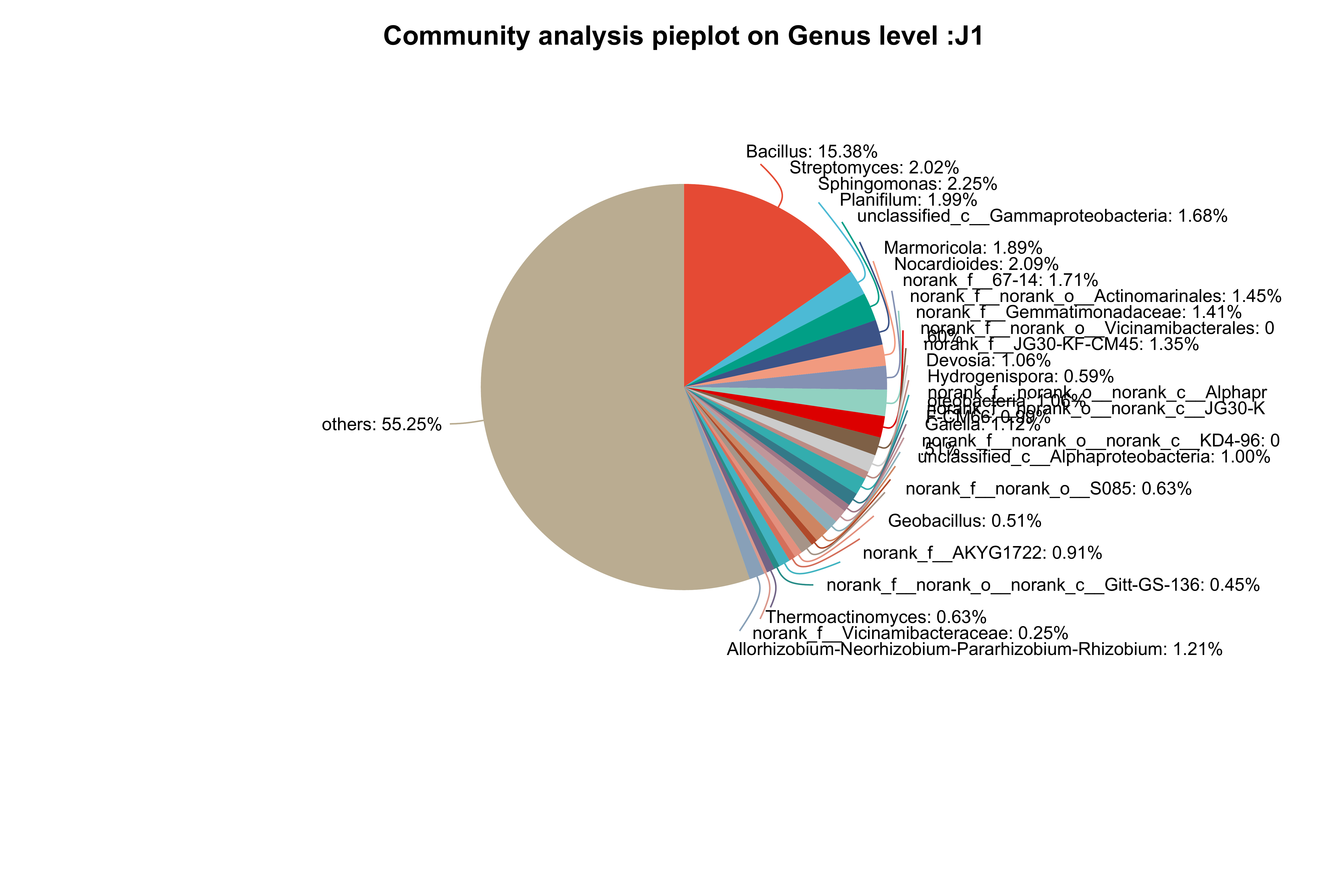

Supplement: Supplementary file 1 [file Data_Sheet_1.zip › Supplementary Figures/FigureS4-A1.jpg]

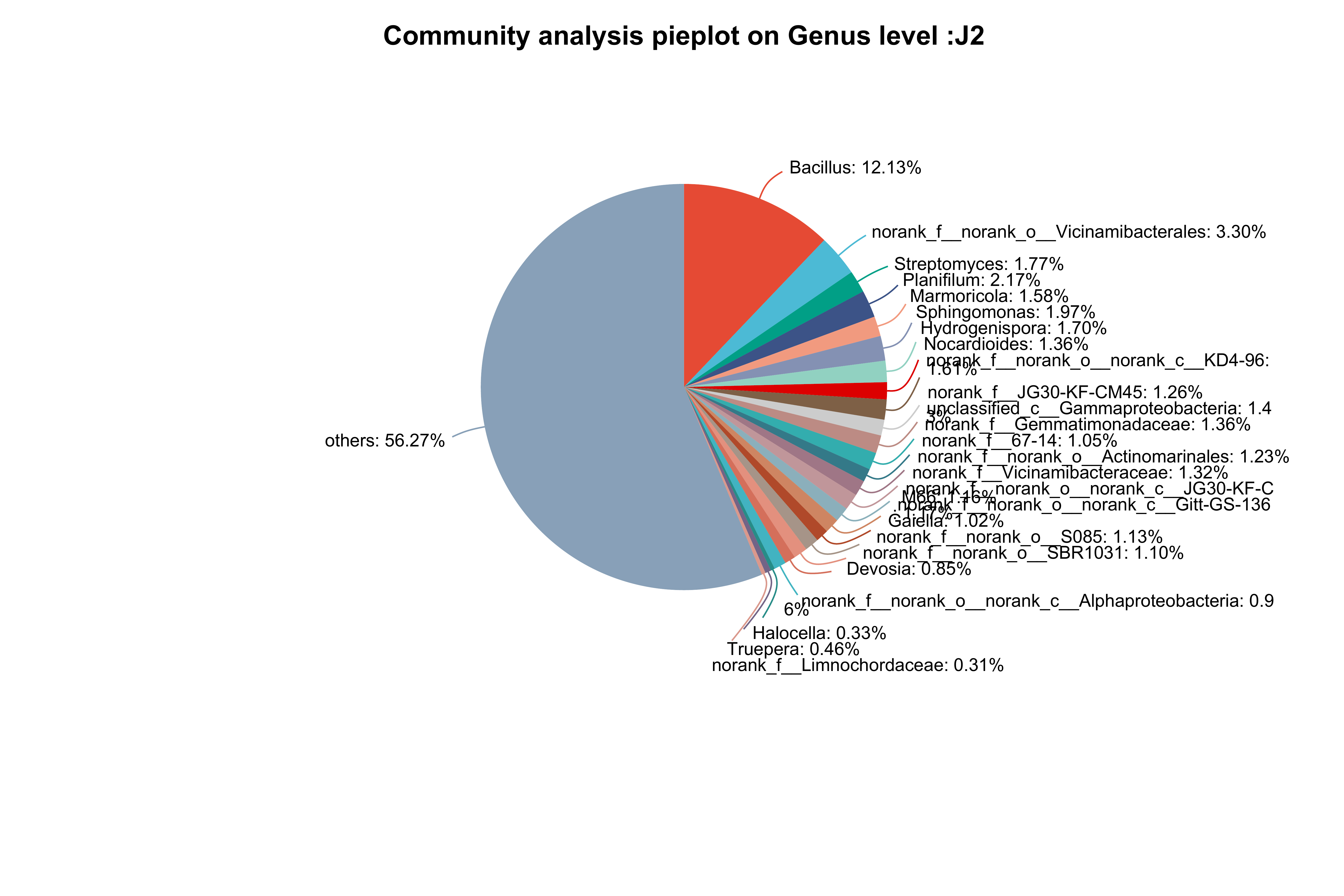

Supplement: Supplementary file 1 [file Data_Sheet_1.zip › Supplementary Figures/FigureS4-A2.jpg]

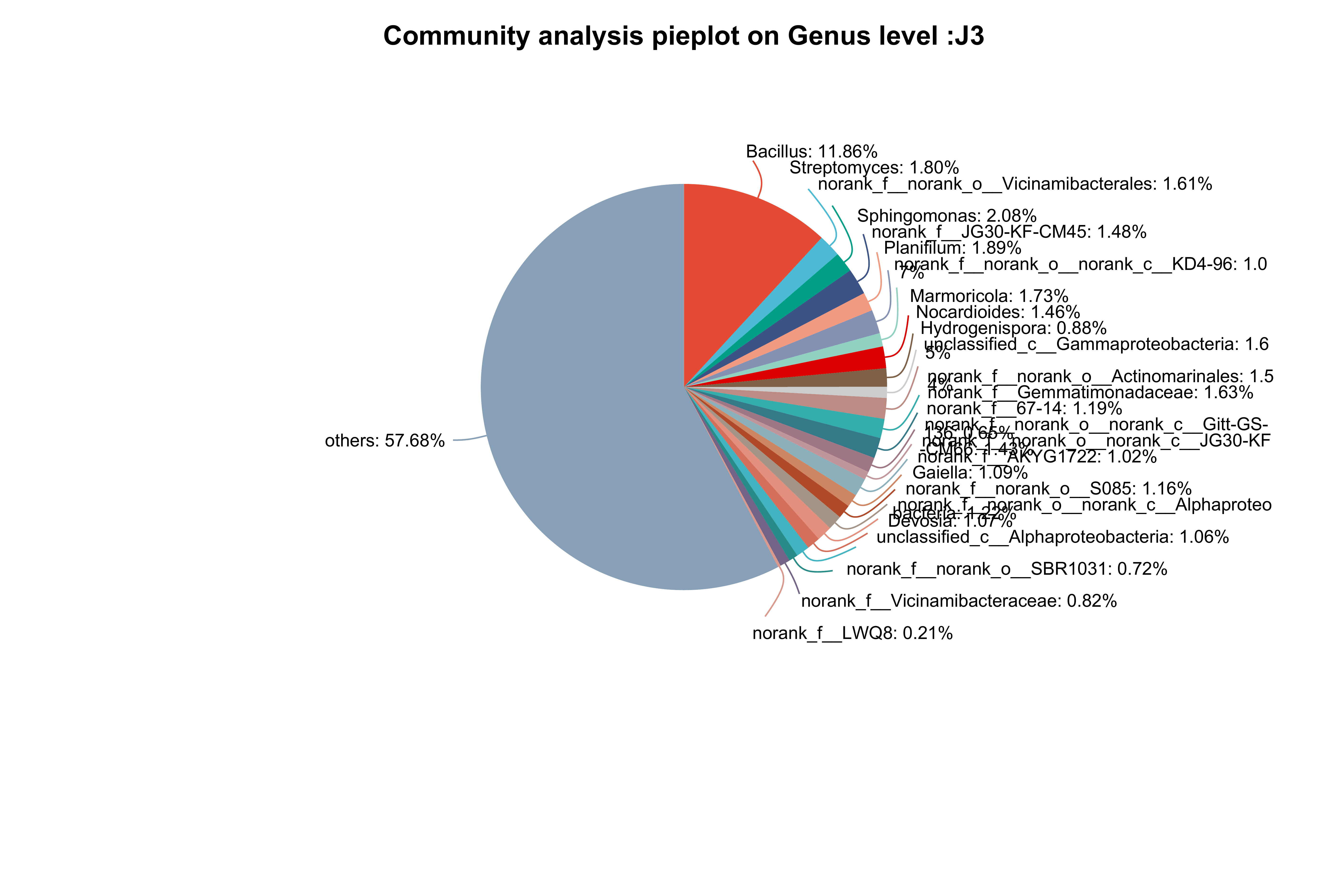

Supplement: Supplementary file 1 [file Data_Sheet_1.zip › Supplementary Figures/FigureS4-A3.jpg]

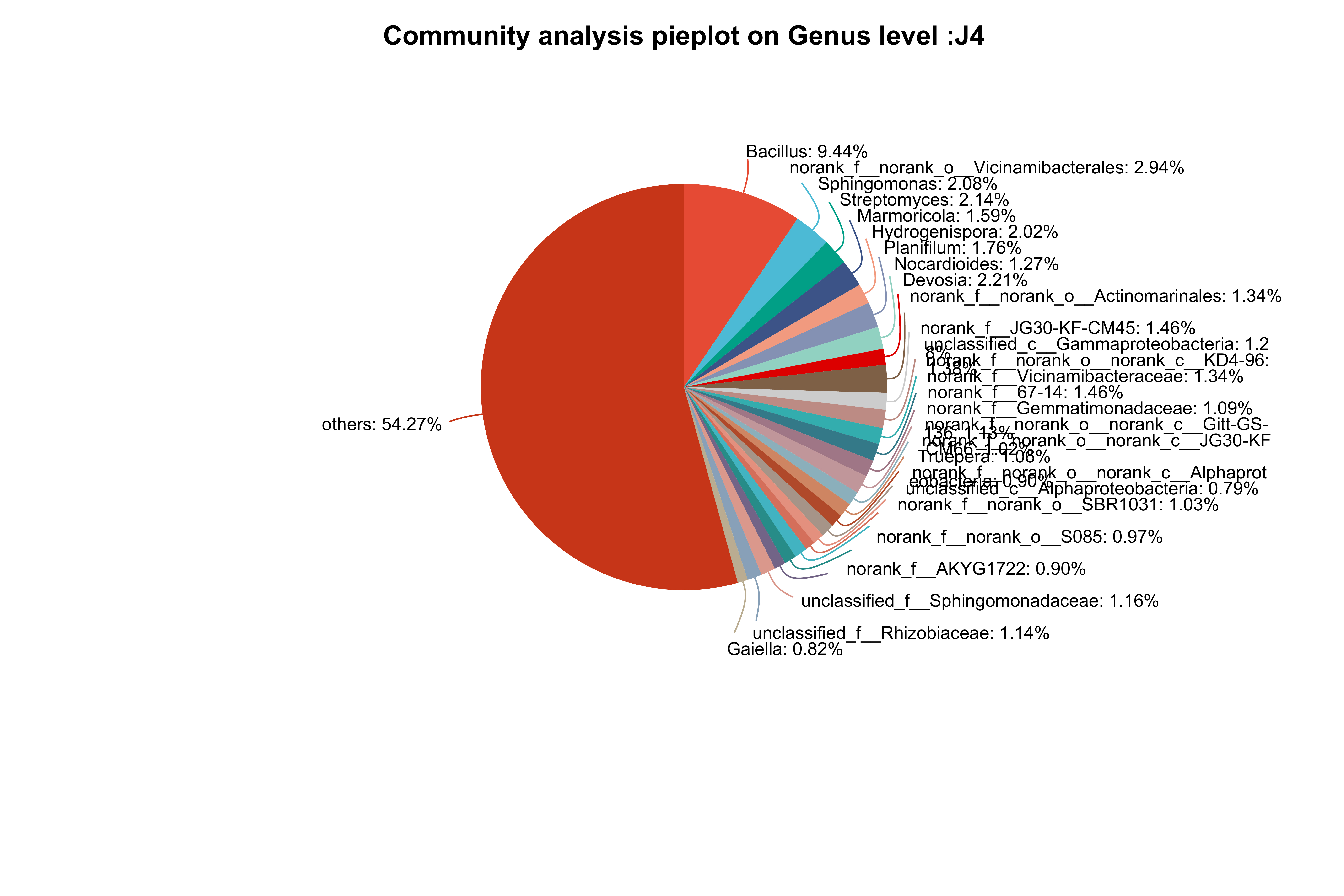

Supplement: Supplementary file 1 [file Data_Sheet_1.zip › Supplementary Figures/FigureS4-A4.jpg]

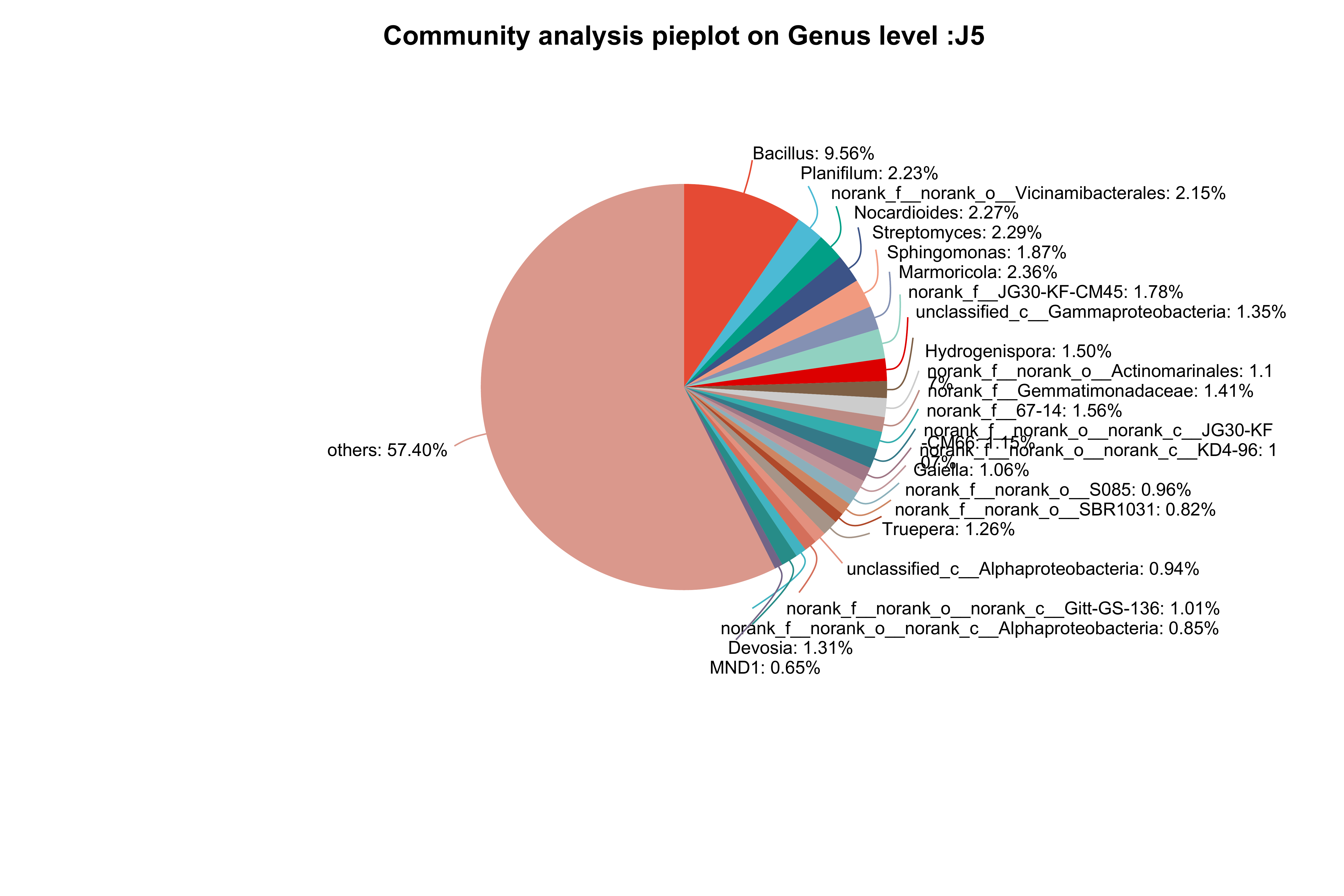

Supplement: Supplementary file 1 [file Data_Sheet_1.zip › Supplementary Figures/FigureS4-A5.jpg]

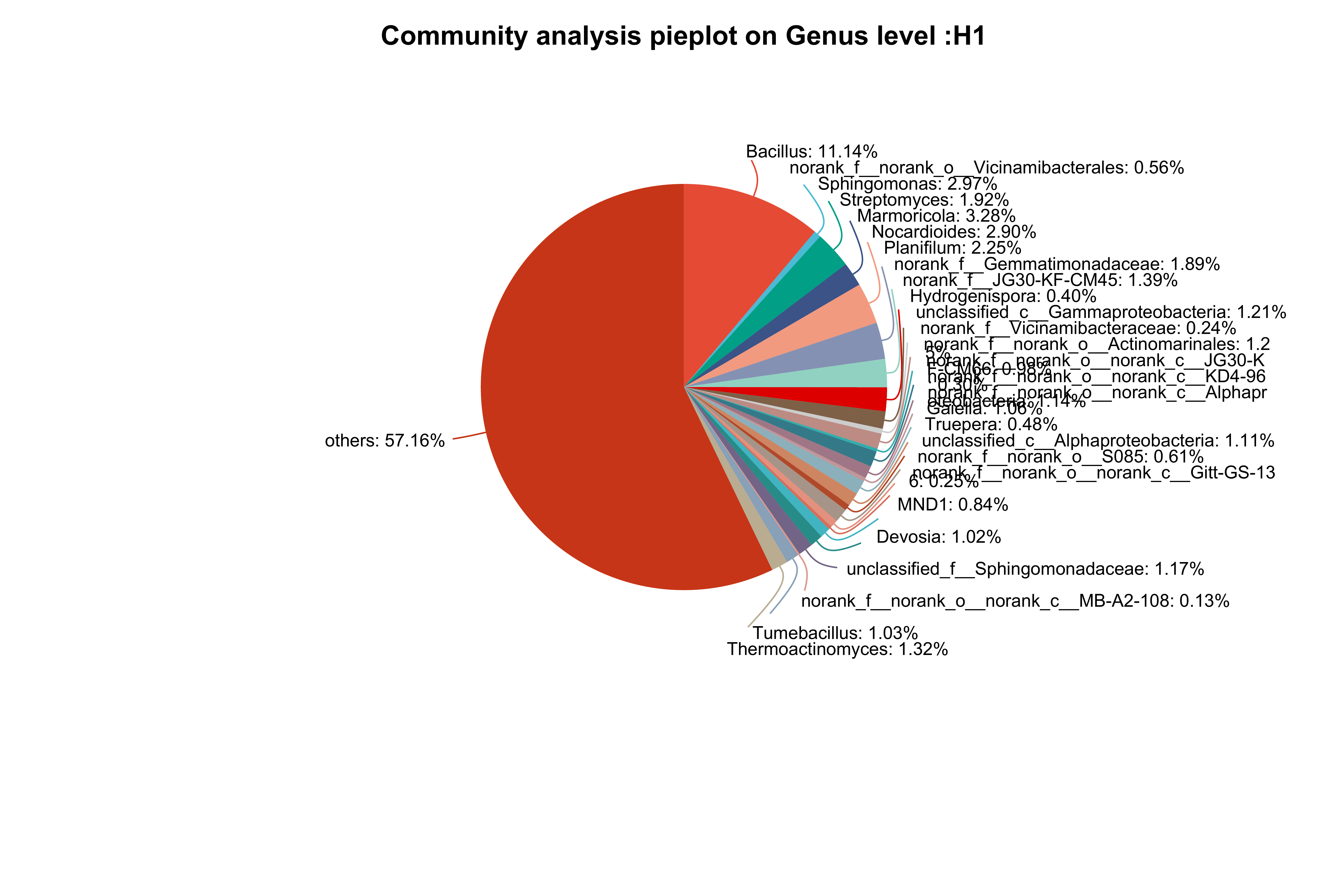

Supplement: Supplementary file 1 [file Data_Sheet_1.zip › Supplementary Figures/FigureS4-B1.jpg]

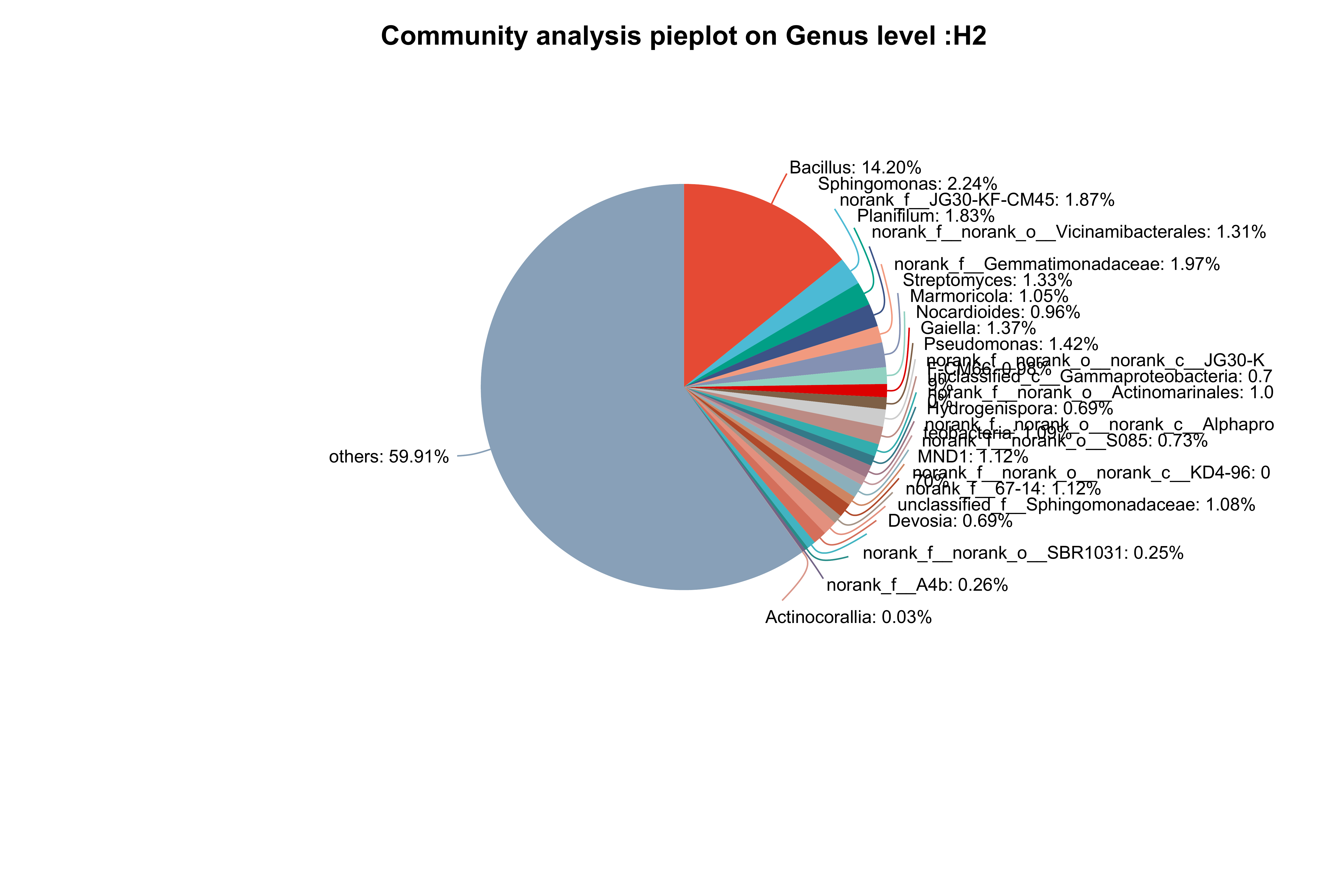

Supplement: Supplementary file 1 [file Data_Sheet_1.zip › Supplementary Figures/FigureS4-B2.jpg]

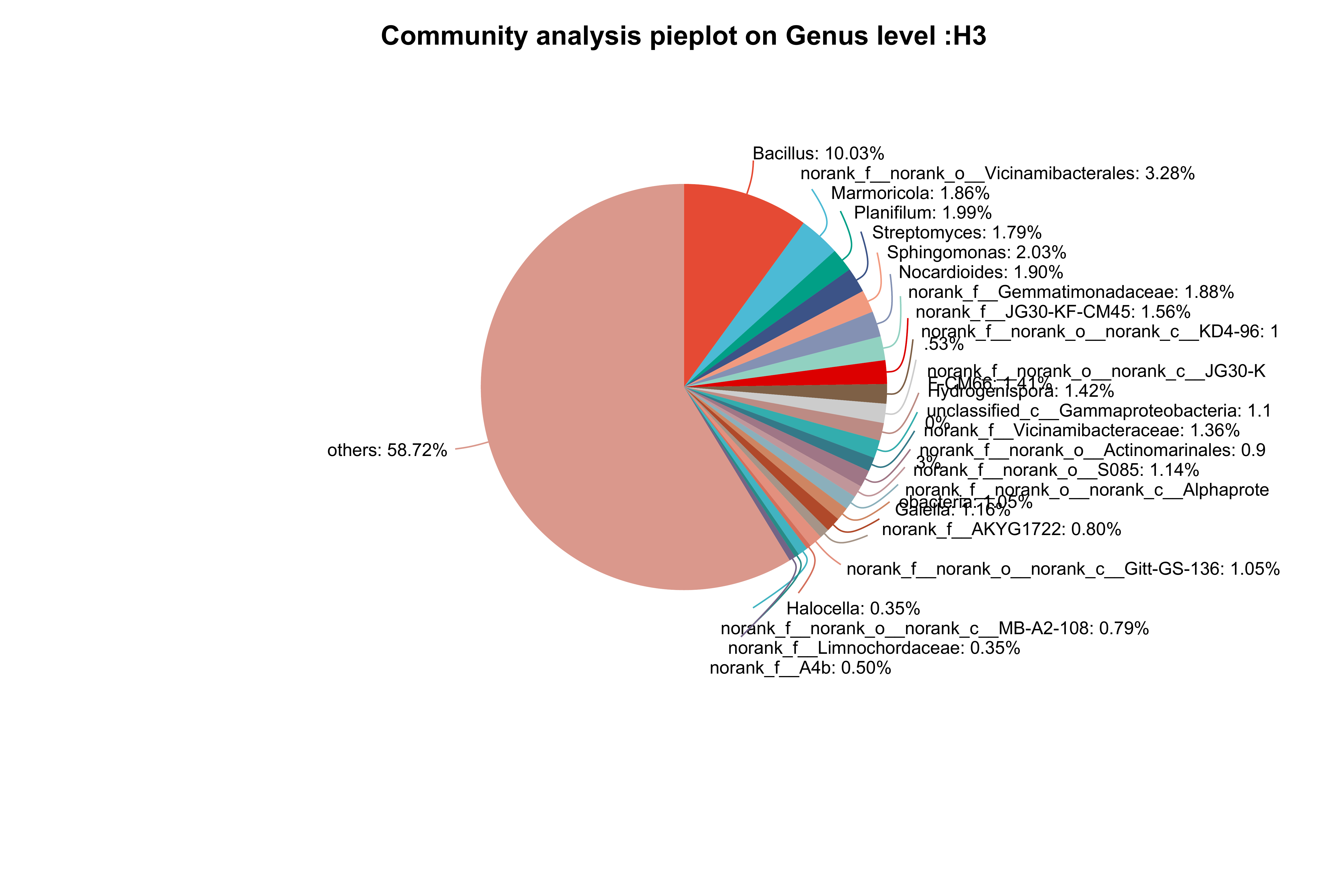

Supplement: Supplementary file 1 [file Data_Sheet_1.zip › Supplementary Figures/FigureS4-B3.jpg]

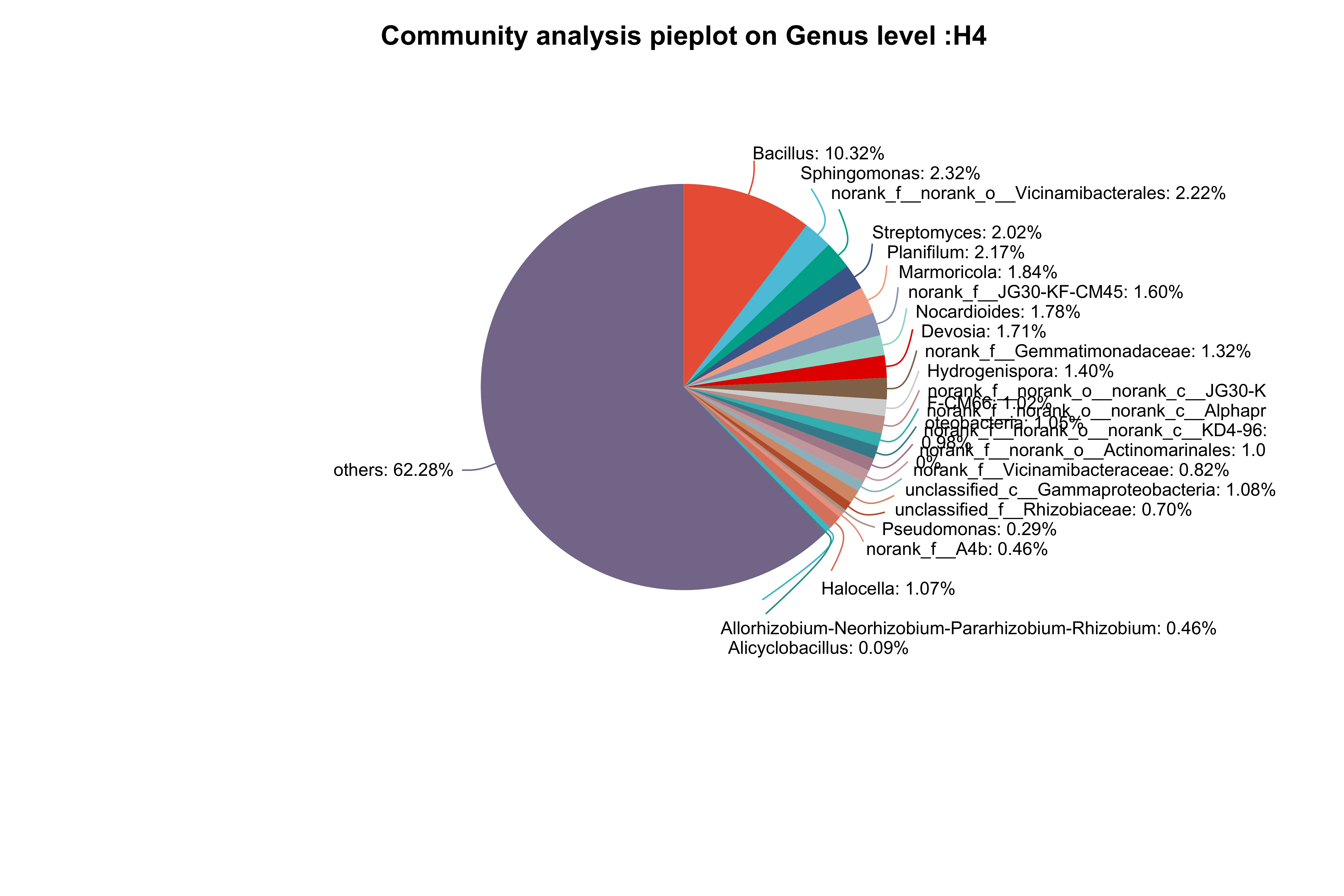

Supplement: Supplementary file 1 [file Data_Sheet_1.zip › Supplementary Figures/FigureS4-B4.jpg]

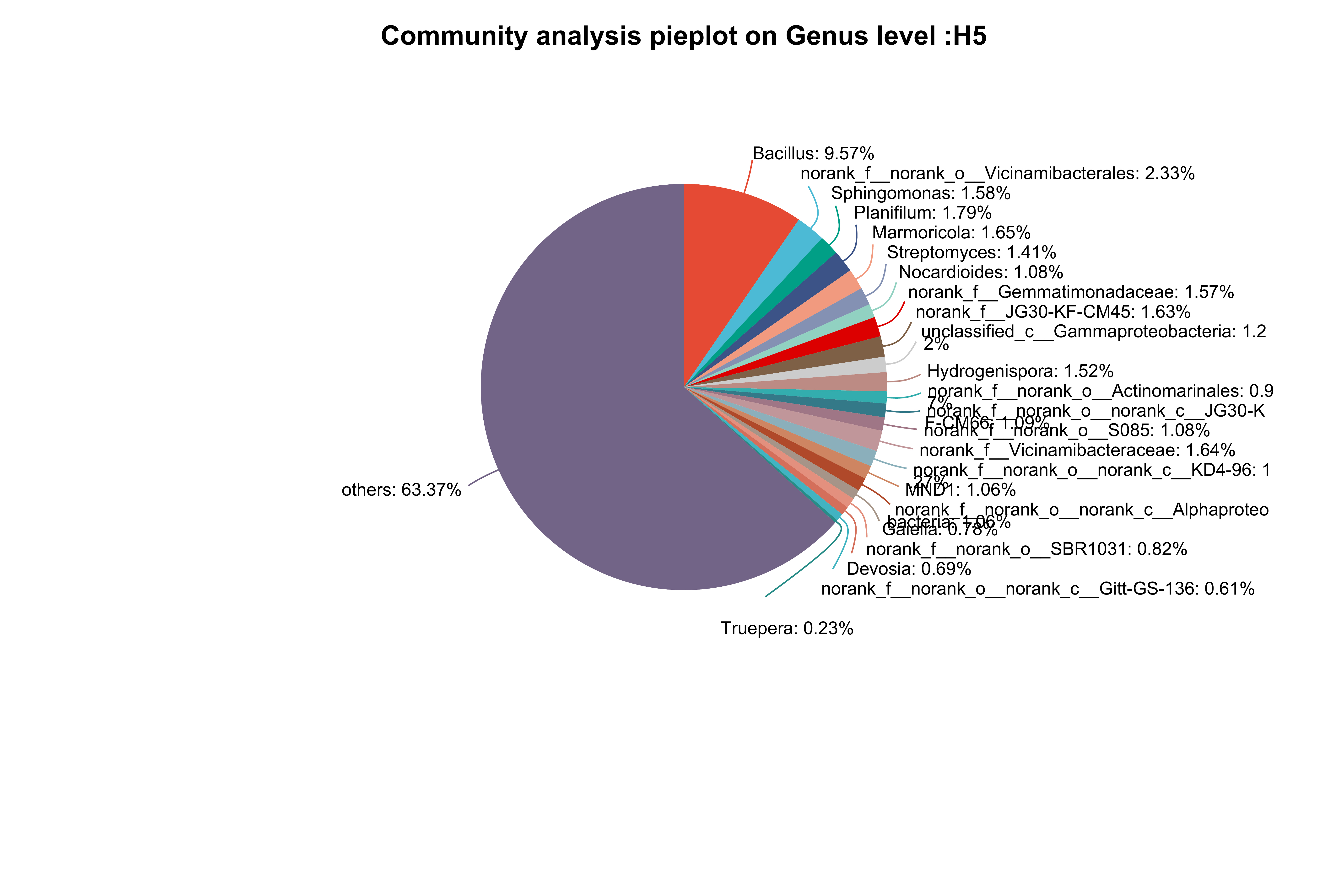

Supplement: Supplementary file 1 [file Data_Sheet_1.zip › Supplementary Figures/FigureS4-B5.jpg]
